# Polyetheretherketone and Its Composites for Bone Replacement and Regeneration

**DOI:** 10.3390/polym12122858

**Published:** 2020-11-29

**Authors:** Chengzhu Liao, Yuchao Li, Sie Chin Tjong

**Affiliations:** 1 Department of Materials Science and Engineering, Southern University of Science and Technology, Shenzhen 518055, China; liaocz@sustech.edu.cn; 2 Department of Materials Science and Engineering, Liaocheng University, Liaocheng 252000, China; 3 Department of Physics, City University of Hong Kong, Tat Chee Avenue, Kowloon, Hong Kong, China

**Keywords:** polyetheretherketone, hydroxyapatite, composite, implant, osteoblast, bone, biocompatibility, elastic modulus, carbon fiber, scaffold

## Abstract

In this article, recent advances in the development, preparation, biocompatibility and mechanical properties of polyetheretherketone (PEEK) and its composites for hard and soft tissue engineering are reviewed. PEEK has been widely employed for fabricating spinal fusions due to its radiolucency, chemical stability and superior sterilization resistance at high temperatures. PEEK can also be tailored into patient-specific implants for treating orbital and craniofacial defects in combination with additive manufacturing process. However, PEEK is bioinert, lacking osseointegration after implantation. Accordingly, several approaches including surface roughening, thin film coating technology, and addition of bioactive hydroxyapatite (HA) micro-/nanofillers have been adopted to improve osseointegration performance. The elastic modulus of PEEK is 3.7–4.0 GPa, being considerably lower than that of human cortical bone ranging from 7–30 GPa. Thus, PEEK is not stiff enough to sustain applied stress in load-bearing orthopedic implants. Therefore, HA micro-/nanofillers, continuous and discontinuous carbon fibers are incorporated into PEEK for enhancing its stiffness for load-bearing applications. Among these, carbon fibers are more effective than HA micro-/nanofillers in providing additional stiffness and load-bearing capabilities. In particular, the tensile properties of PEEK composite with 30wt% short carbon fibers resemble those of cortical bone. Hydrophobic PEEK shows no degradation behavior, thus hampering its use for making porous bone scaffolds. PEEK can be blended with hydrophilic polymers such as polyglycolic acid and polyvinyl alcohol to produce biodegradable scaffolds for bone tissue engineering applications.

## 1. Introduction

**Figure 1:**
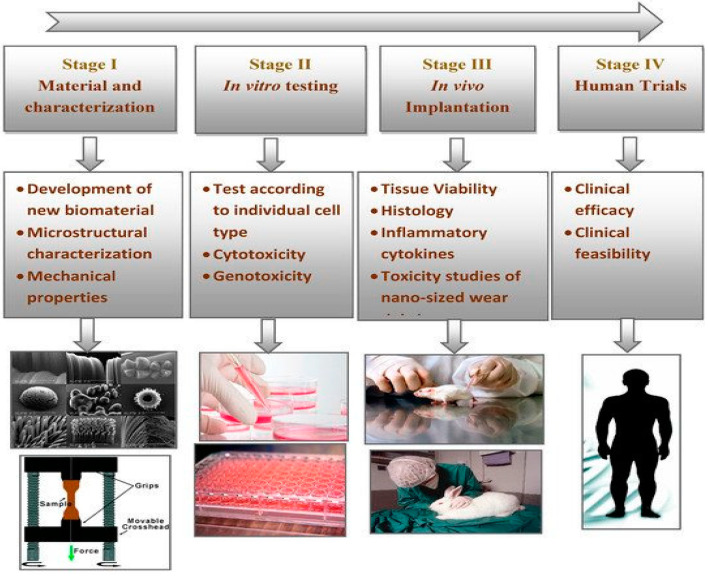
Multiple stages involved in the assessment of newly developed biomaterials for bone tissue engineering. Reproduced from [2] with permission of MDPI under the conditions of Creative Commons Attribution license.

Bone fractures and segmental bone defects pose a significant risk of patient morbidity and economic burden to the public healthcare system [1]. With increasing numbers of geriatric population and people suffering from traffic and sports injuries, bone cancer and disorders, dental defects and congenital disorders, the demands for hip replacement, craniomaxillofacial and spinal implants are rising globally in recent years. In this perspective, the development of novel biomaterials for hard tissue replacements is considered of technological importance. Biomaterials for medical devices should be non-toxic to cells, having remarkable biocompatibility and bioactivity for the regeneration of bone tissues. Osseointegration of medical implants with the surrounding tissues plays a decisive role for new bone regeneration and healing in maxillofacial reconstruction, spinal fusion and hip replacement applications. In general, the development and successful implementation of novel biomaterials for bone tissue engineering applications involve several stage procedures including proper selection and fabrication of materials, microstructural and mechanical characterizations, in vitro cellular tests and in vivo animal models, and final clinical trials (Figure 1) [2]. 

Biomaterials used in bone tissue engineering generally include ceramics, metals, polymers and composites. Bioceramics like calcium phosphates are well known bioactive materials that mimic and integrate with human bones by inducing an apatite layer on their surfaces [3,4]. However, brittle CaP-bioceramics exhibit low fracture toughness, thereby confining their use as coating materials for metallic implants. Metallic materials with excellent mechanical strength and ductility, e.g., titanium alloy, cobalt chrome molybdenum alloy and 316L stainless steel are commonly used for making load-bearing bone replacements, dental implants, spinal fusion and bone fixation devices [5,6,7]. However, metallic implants have certain drawbacks including stress shielding effect and corrosion issues. The former derives from a significant mismatch in elastic modulus between the implant and host bone, leading to the reduction of bone mass and implant loosening [8]. The elastic modulus, tensile strength and elongation at fracture of human cortical bone range from 7–30 GPa, 50–150 MPa and 1–3%, respectively [9]. The elastic moduli of CoCrMo alloy, austenitic stainless steel and Ti-6Al-4V alloy are reported to be 200–230, 200 and 110 GPa, respectively [10]. So, the modulus of metallic alloys is remarkably higher than that of cortical bone, resulting in stress shielding and implant failure. Corrosion is due to the release of metal ions through the interaction of implants with body fluid. In particular, released Ni^2+^, Cr^3+^ and Co^2+^ ions from the CoCrMo alloy and 316L stainless steel would cause allergy, inflammation and cytotoxicity [11,12]. Moreover, chloride ions in human body fluid induce pitting corrosion on those alloys due to the breakdown of passive films [11,13,14]. 

Polymers with facile processability, good chemical resistance and light weight are attractive materials for orthopedic applications [15,16,17,18,19,20]. Biodegradable polymers with poor mechanical strength such as polylactic acid, polyglycolic acid, and their copolymers are generally employed for making scaffolds for tissue engineering applications [15,16]. The biocompatibility and mechanical performance of biodegradable polymers can be enhanced by adding bioactive nanofillers [21,22,23]. Non-degradable polymers such as polyethylene and polyetheretherketone (PEEK) find applications in orthopedics where long-term stability is needed. High-density polyethylene (HDPE) is typically used for tendon repair and catheter tube, while ultra-high molecular weight polyethylene (UHMWPE) is employed as a bearing material in total joint prostheses [24]. HDPE is very ductile with an elastic modulus of 1.3 GPa [25]. Its mechanical strength and stiffness can be improved by reinforcing with hydroxyapatite (HA) having a stiffness of 120.6 GPa [26]. Hydroxyapatite [Ca_10_(PO_4_)_6_(OH)_2_] is a bioceramic having compositional similarity with the mineral component of human bones and teeth. Human bone tissue is a biocomposite consisting of 70% nano-hydroxyapatite (nHA) platelets and 30% collagen fibrils. To mimic the structural component of human bones, HAPEX^TM^ composite has been developed for bone replacement by incorporating 40vol% HA into the HDPE matrix [25]. However, Young’s modulus of HAPEX^TM^ (4.4 GPa) is considerably lower than that of cortical bone, rendering its application for non-load bearing maxillofacial implants only. 

PEEK is a semicrystalline polymer with an aromatic backbone chain connecting by the ketone and ether groups. PEEK is considered as a high-performance polymer due to its excellent chemical resistance, high melting temperature (340 °C), superior radiation and sterilization resistance, high modulus of elasticity (3.7–4.0 GPa) and tensile strength (103 MPa). Moreover, PEEK is a radiolucent material, facilitating radiographic assessment of the implants [27,28]. In this respect, PEEK has been successfully used as a biomaterial for spinal fusion, cranial reconstruction and dental implant applications [28,29,30,31,32,33,34,35,36,37,38]. In spite of these advantages, however, bioinert PEEK is unfavorable for osteoblastic cell adhesion. As such, HA microparticles, HA whiskers and other ceramic particulates are incorporated into PEEK for enhancing bone cell adhesion and mechanical stiffness [39,40,41]. For example, Abu Bakar et al. added 5–40 vol% HA microparticles (mHA) with a mean size of 25.68 µm to PEEK. In vivo pig model revealed that 20 vol% mHA fillers promote bone regeneration for PEEK. However, the tensile strength of PEEK decreased markedly from 80 MPa to 58 and 44 MPa by adding 20 and 40 vol% mHA, respectively [39]. Thus, the composites had lower tensile strength than pure PEEK, i.e., the fillers carried no applied load during tensile testing. This was attributed to the fracture of large mHA fillers and poor interfacial mHA-PEEK bonding. To enhance the stiffness and mechanical strength of PEEK, continuous and discontinuous carbon fibers (CFs) with high elastic modulus were added to PEEK accordingly [42]. The PEEK/CF composites have been used for lumbar interbody fusion cages, acetabular cup of hip prostheses, and dental implants/abutments [43,44,45,46,47].

In recent years, advances in nanotechnology have greatly contributed to the creation of novel nanomaterials with functional properties for biomedical applications [48,49]. Nanomaterials with small size and large surface area exhibit superior chemical, mechanical and physical properties compared to their micron-sized system [50,51,52,53]. As an example, nanohydroxyapatite (nHA) has shown improved bone cell adhesion over mHA [54,55,56,57,58,59]. Thus, nHA is incorporated into both degradable and non-degradable polymers for enhancing osteoblastic adhesion and proliferation [60,61,62,63,64,65,66,67,68,69]. Apart from nHA, carbonaceous nanofillers of different dimensions such as carbon nanotubes (CNTs), carbon nanofibers (CNFs) and graphene oxide sheets also promote the adhesion, growth and differentiation of osteoblasts [70,71,72,73,74,75,76,77,78,79,80,81]. As a result, carbon-based nanomaterials are also incorporated into polymers for enhancing their biocompatibility in many tissue engineering applications [61,62,63,82,83,84,85,86]. Recently, Ma et al. fabricated PEEK/nHA nanocomposites by means of vibrating ball-mill mixing and injection molding [87]. By adding 40 wt % (21.5 vol%) nHA, Young’s modulus of PEEK/nHA reaches 4.6 GPa, being considerably smaller than the lower limit of the modulus of cortical bone, i.e., 7 GPa. Tjong and coworkers melt-compounded PEEK/nHA nanocomposites in an extruder followed by injection molding. The Young’s modulus of PEEK/21.5 vol% nHA nanocomposite exceeds that of cortical bone, i.e., 7.85 GPa [62]. However, this material is extremely brittle with a low tensile elongation of 0.63%. To tackle the issue of brittleness, low loading levels of CNFs or CNTs are added to PEEK/nHA nanocomposites [62,63]. This article gives a comprehensive and updated review of studies relating the development, fabrication, biocompatibility and mechanical properties of PEEK and its composites for hard and soft bone tissue engineering applications. 

## 2. PEEK Implants

**Figure 2:**
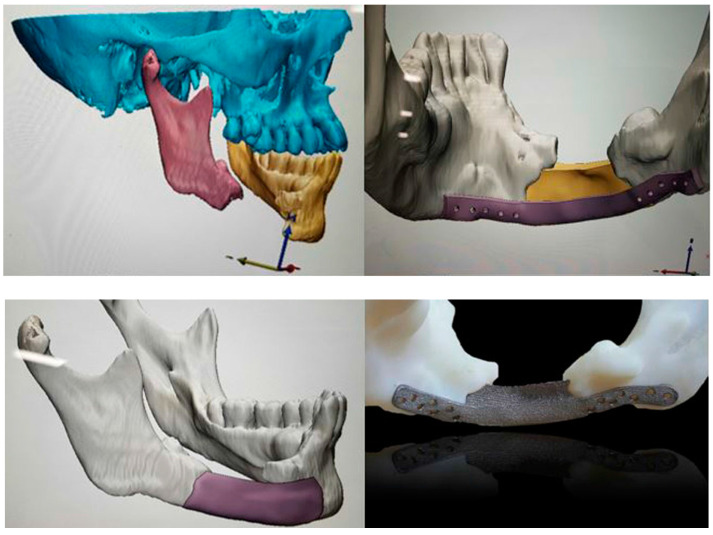
The design and fabrication of polyetheretherketone patient-specific implants using CAD/CAM system for jaw reconstruction. Reproduced from [31] with permission of Springer under the terms of the Creative Commons Attribution 4.0 International license.

PEEK has been widely employed as spinal implant materials including cages, rods, and screws to maintain rigid stability in the spine such that the bones can fuse together gradually [28,29,30,31,88,89]. PEEK spinal fusions can prevent serious problems of stress shielding and vertebral collapse typically encountered by metallic materials. PEEK can also be used to fabricate patient specific implants (PSIs) for reconstructing maxillofacial deformities [31,34,90,91,92]. PEEK PSIs demonstrate higher clinical efficacy in restoring the shape of the damaged orbit [90]. Recently, additive manufacturing (AM) has emerged as an effective tool for printing complex objects for biomedical applications [93,94,95]. AM technology is particularly useful to make patient-specific cranial implants using a computer aided design (CAD)/computer aided manufacturing (CAM) software. In the process, the skull of a patient is scanned by computer tomography, and the data are delivered to a CAD/CAM system for manufacturing three-dimensional (3D) PEEK PSIs (Figure 2). Reliable clinical results for PEEK PSIs in maxillofacial surgery have been reported by Jarvinen et al. very recently [91]. Meanwhile, Bagul et al. and Serra et al. have printed 3D PEEK lumbar cages using fused filament fabrication (FFF), or generally known as fused deposition modeling (FDM). FFF is one of the AM techniques which employs polymer filaments created by the hot melt extrusion for printing 3D medical implants layer-by-layer. The 3D printed cages are capable of providing mechanical strength for lumbar cage applications [94].

In general, poor osseointegration at the bone-implant interface would lead to a failure of the medical devices. The biological interface between a device and the surrounding host tissue plays a key role in the overall performance of implants. Clinical strategies to increase osseointegration and osteoinduction of orthopedic implants still remain a big challenge to surgeons, chemists and materials scientists. PEEK-based implants with smooth and hydrophobic surfaces are bioinert, having limited capacity to bind to bone tissues. As a result, several approaches such as the creation of porous feature, surface modification and coating deposition can be used to enhance osseointegration ability of PEEK [30,31,90,96,97,98,99,100,101,102,103,104,105]. Porous features offer a remarkable osteogenic response at the cell level, thereby facilitating bone ingrowth of spinal fixation devices. However, the presence of pores throughout entire PEEK implants would degrade the mechanical strength greatly [106]. In this respect, Torstrick et al. have introduced a porous surface layer of few micrometers thick with high interconnectivity on PEEK using a melt extrusion and porogen (NaCl) leaching process. The porous surface layer facilitates mechanical interlocking of bone, without sacrificing mechanical performance of PEEK. Porous PEEK with a surface porosity of 67.3% and a mean pore size of 279.9 µm retains 73.9% of the strength and 73.4% of the elastic modulus of solid PEEK [91]. Moreover, surface porous PEEK facilitates cell attachment and proliferation of human femoral osteoblasts (hOBs) and human mesenchymal stem cells (hMSCs) [96,97]. The surface porous structure promotes vascularization and bone ingrowth at 6 and 12 weeks after implantation into a rat femoral segmental defect model [91]. On the basis of mechanical characterization, in vitro cellular response and in vivo animal model results, PEEK interbody fusion implant with a solid core and porous structure on the superior and inferior faces has been developed by Vertera Spine (Atlanta, GA, USA) under the tradename COHERE^®^. COHERE^®^ shows a successful clinical use in anterior cervical discectomy and fusion (ACDF) surgery and becomes a popular choice of biomaterial for cervical myelopathy treatment [97]. 

### 2.1. Coatings

**Figure 3:**
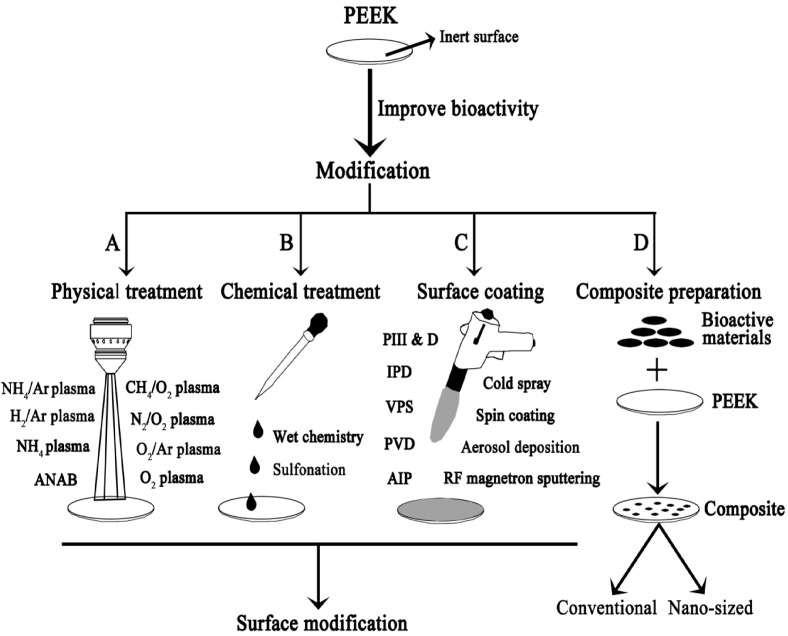
Current strategies typically used to improve bioactivity of PEEK. Reproduced from [101] with permission of MDPI under the terms and conditions of the Creative Commons Attribution license.

**Figure 4:**
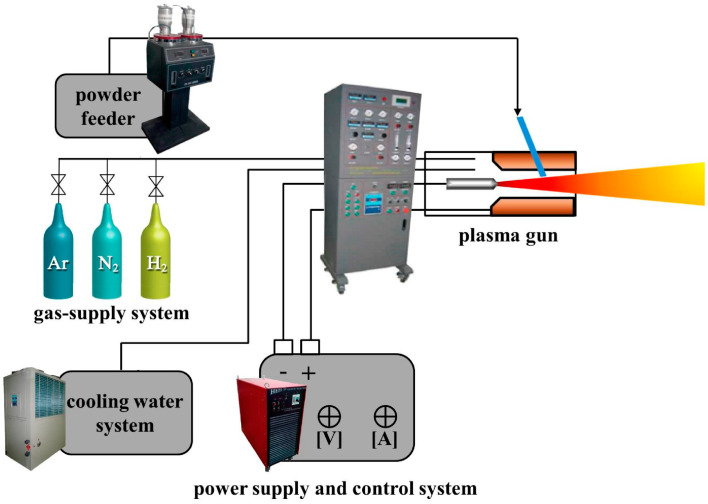
Schematic illustration of air plasma spray set-up. Reproduced from [106] with permission of MDPI under the terms and conditions of the Creative Commons Attribution license.

Deposition of PEEK surface with osteoconductive materials like HA, Ti and titanium dioxide (TiO_2_) can enhance its bioactivity and osseointegration greatly [99,100,101,102,103,104,105]. Those surface coatings can be deposited on PEEK using several techniques including plasma immersion ion implantation (PIII), ionic plasma deposition (IPD), vacuum plasma spraying (VPS), arc ion plating (AIP), cold spraying, spin coating, aerosol deposition, radio-frequency (RF), and magnetron sputtering (Figure 3). Among these, plasma spraying can yield rough and porous coating on PEEK, which is beneficial for bone cell adhesion and ingrowth. The technique involves the use of an arc inside the plasma gun to melt ceramic or metal powders at high temperatures. The molten droplets are then propelled onto the target substrate, followed by flattening, rapid solidification, and cooling to form a porous coating layer (Figure 4) [106]. The spraying process can be performed in a vacuum chamber instead of air at atmospheric pressure to prevent the oxidation of the coating material, which is referred to as ‘vacuum plasma spraying’. 

#### 2.1.1. Hydroxyapatite-Coated PEEK Implants

Vacuum plasma spraying is capable of forming rough and porous bioceramic coatings on PEEK for promoting in-growth of bone cells into the coatings. The process is carried out at high temperatures, typically at about 2000 °C or above for melting HA with a melting temperature of 1670 °C. However, inhomogeneous melting of HA into tricalcium phosphate (Ca_3_(PO_4_)_2_) and tetracalcium phosphate (Ca_4_O(PO_4_)_2_) during plasma spraying would deteriorate the performance of deposited coatings [107]. To prevent nonuniform HA formation and thermal damage of polymer substrate under high temperature of plasma, cold spraying can be employed to deposit HA layer on PEEK at lower temperatures. The cold spray process utilizes the energy stored in high pressure compressed gas to accelerate powder particles at very high velocities, ca. 300–1200 m/s. The particles with high kinetic energy impact on the target without melting, forming a homogenous surface coating accordingly. For instance, Lee et al. coated HA on PEEK using cold spraying process. The HA-PEEK samples promoted the differentiation of bone marrow-derived mesenchymal stem cells as manifested by enhanced alkaline phosphatase activity, calcium production, and bone sialoprotein (BSP) expression [105]. Furthermore, the insertion of HA-PEEK cage implant into minipig model showed the regeneration of bone tissues. 

**Figure 5:**
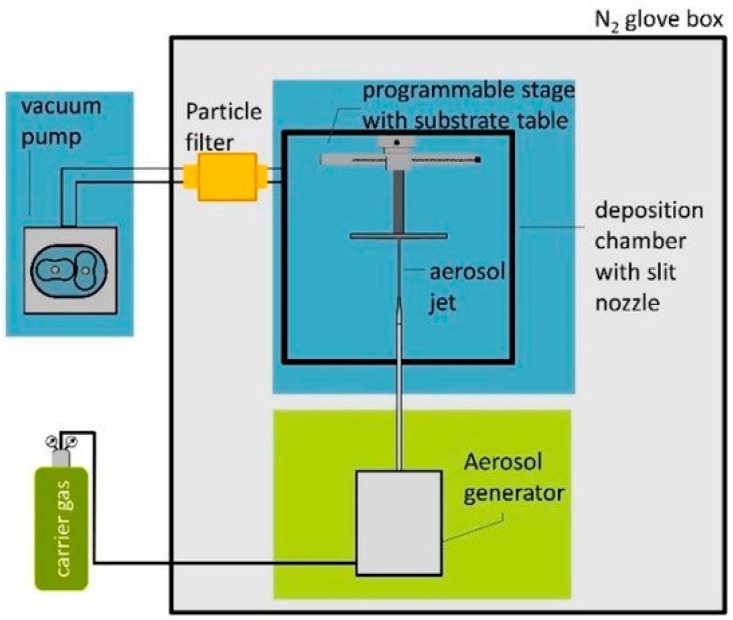
Schematic illustration of typical coating formation using aerosol deposition. Reproduced from [108] with permission of MDPI under the terms and conditions of the Creative Commons Attribution license.

Alternatively, aerosol deposition can also effectively deposit ceramic powders on the polymer substrates to form coatings at lower temperatures. Aerosol deposition is a spray coating process using an aerosol generator in which the carrier gas and ceramic powders are contained in a vibrating chamber. The aerosolized particles are then transported to the deposition chamber with a slit nozzle to impact them on the target material at velocities between 100 and 600 m/s (Figure 5) [108]. Hahn et al. coated PEEK with an HA layer by means of aerosol deposition followed by hydrothermal annealing for enhancing the crystallinity of HA [109]. As a result, HA-PEEK exhibited excellent biocompatibility under in vitro cell culture and in vivo animal model tests. Rabiei and Sandukas deposited HA on PEEK using RF magnetron sputtering. Prior to HA deposition, an yttria-stabilized zirconia (YSZ) layer was first coated on the PEEK substrate to provide heat shielding. The as-deposited HA layer exhibited an amorphous structure. Then HA/YSZ coated PEEK was subjected to microwave annealing in order to form crystalline HA [110]. Cell cultivation tests revealed a marked increase in initial bone cell attachment and proliferation on microwave-annealed samples, compared with uncoated PEEK and PEEK with amorphous HA surface layer. In another study, Rabiei and coworkers used ion beam assisted deposition (IBAD) to form HA/YSZ coatings on cylindrical PEEK implants. Thereafter, microwave heating with and without subsequent autoclaving were employed to crystallize amorphous HA layer [111]. The HA/YSZ-PEEK samples were implanted into lateral femoral condyle of the rabbits. The heat-treated HA/YSZ-PEEK samples were found to exhibit improved osseointegration, higher bone regeneration and bone-implant contact area when compared to pure PEEK. 

**Figure 6:**
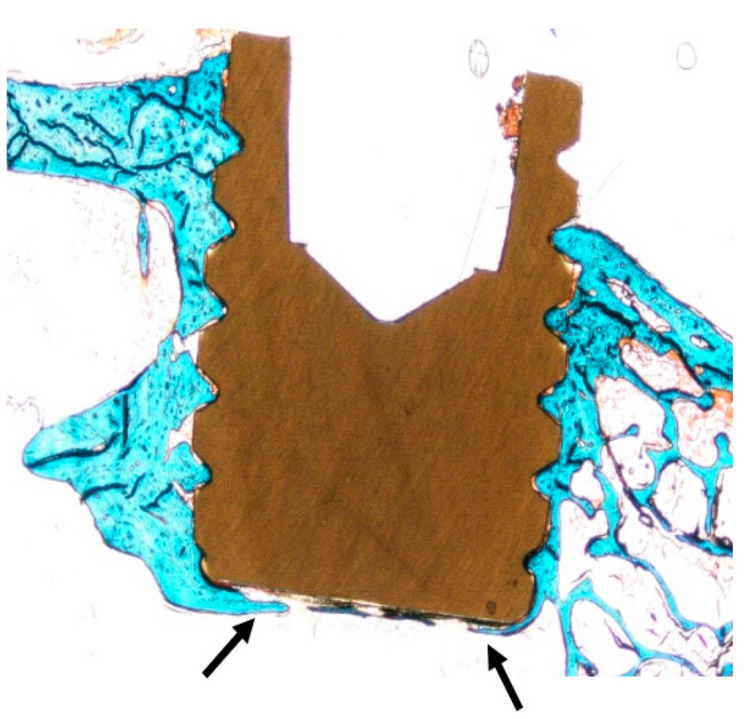
Histologic section of nHA-coated PEEK screw implanted into the rabbit tibia for 12 weeks. The threads are filled with bone tissues, showing osteoconductive feature of nHA-coated PEEK (arrow). Reprinted from [104] with permission of MDPI under the terms and conditions of the Creative Commons Attribution license.

Recently, Johansson et al. reported that spin-coated nHA-PEEK screws exhibit a remarkable improvement in osteogenic properties. The nHA-PEEK screws were implanted into the tibia and femur of rabbits for 3–12 weeks (Figure 6) [103,104]. The nHA-PEEK after 3 weeks showed osseointegration as revealed by higher bone implant contact (BIC) and total bone area percentage values compared to uncoated PEEK. After 12 weeks of implantation, BIC was significant enhanced in femur and tibia around HA-PEEK screws compared with pure PEEK [103,104]. 

#### 2.1.2. Ti-Coated PEEK Implants

Hydrophobic PEEK surface often reduces cellular adhesion and growth. Thus, implant surfaces with adequate hydrophilicity favor cell adhesion and enhance interactions with surrounding tissues. In this perspective, PEEK can be coated with a titanium surface layer for improving its wettability, resulting in better cell attachment and biocompatibility [112,113,114,115]. For instance, Han et al. coated Ti onto PEEK using electron beam deposition. The Ti layer adhered firmly to the PEEK substrate and improved its wettability [112]. As such, modified PEEK surface promoted the adhesion of mouse osteoblastic cell line (MC3T3-E1). Moreover, in vivo animal tests revealed that Ti-PEEK implants have a higher BIC ratio than pure PEEK implants.

**Figure 7:**
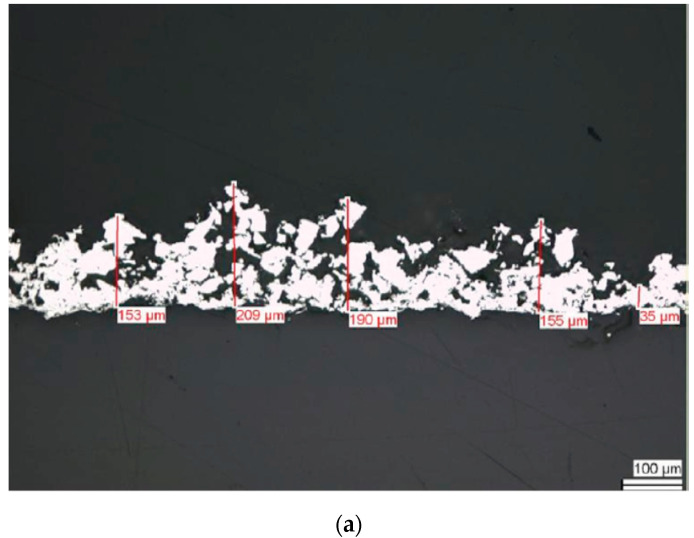

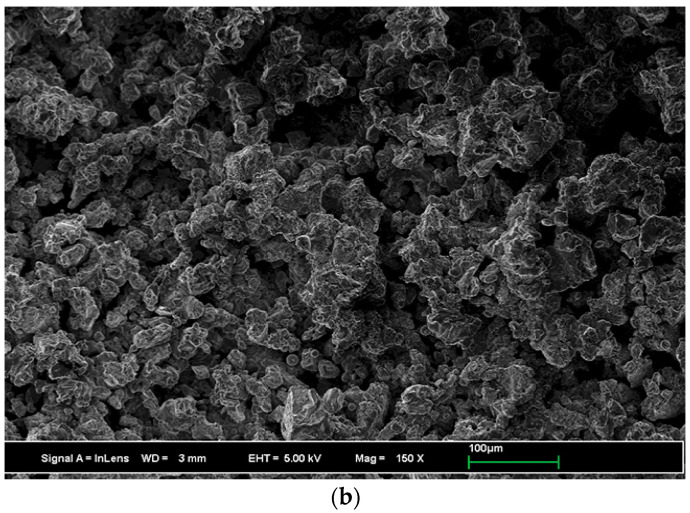
Scanning electron microscopic images showing (**a**) cross-sectional and (**b**) top views of porous Ti layer coated on PEEK through vacuum plasma spraying. Reproduced from [113] with permission of Dove Press under the Creative Commons Attribution—Non-Commercial (unported, v3.0) license.

**Figure 8:**
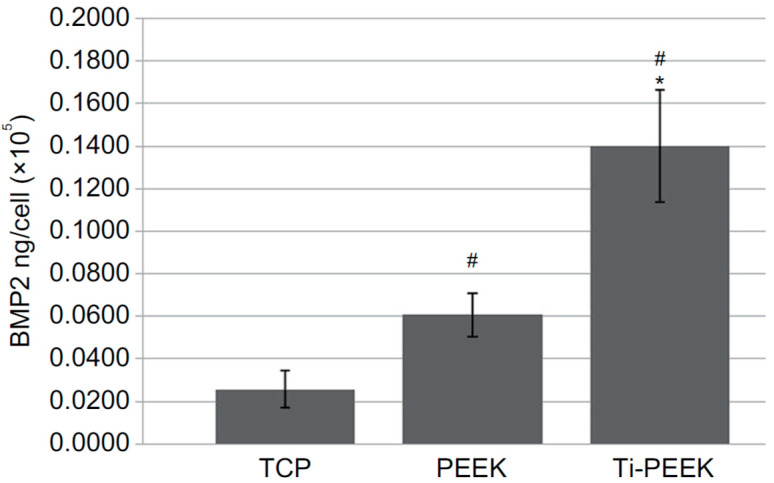
BMP-2 protein levels secreted by MG63 cells cultivated on Ti-PEEK, PEEK, and TCP surfaces. TCP: tissue culture plastic. * denotes *p* < 0.05 vs PEEK; # implies *p* < 0.05 vs TCP. Reproduced from [113] with permission of Dove Press under the Creative Commons Attribution—Non-Commercial (unported, v3.0) license.

Very recently, Cheng et al. studied in vitro cell culture and in vivo sheep model of Ti-PEEK disks prepared by vacuum plasma spraying [113]. The plasma sprayed Ti coating is rough and porous (Figure 7a,b). Rough Ti-PEEK disks induce the adhesion and proliferation of osteoblasts and upregulate the gene expressions including alkaline phosphatase (ALP) and bone morphogenetic protein-2 (BMP-2) [116]. ALP activity is considered as an early marker of cell differentiation; BMP-2- is the growth factor that promotes osteoblastic cell differentiation [117,118]. From the literature, human osteoblast-like osteosarcoma cells (MG63) cultured on the surfaces with increasing roughness show decreased proliferation and increased cell differentiation [119]. Cheng et al. also reported a reduced proliferation, but high ALP and BMP-2 levels of MG63 on Ti-PEEK (Figure 8). In vivo animal tests showed that Ti-PEEK samples exhibit enhanced osseointegration and increased bone formation compared to unmodified PEEK. Biomechanical pullout measurement revealed that Ti-PEEK samples have higher pullout force after 12 and 24 weeks of implantation. The higher pullout strength derived from mechanical interlocking between the implant and bone. Similarly, Walsh et al. demonstrated that the titanium coating significantly enhanced the shear strength at the bone-implant interface of plasma sprayed Ti-PEEK after 4 weeks of implantation into cortical bone of adult sheep. Furthermore, the shear strength of Ti-PEEK continued to increase with time compared to PEEK [115]. 

It is noted that titanium coating deposited on PEEK is susceptible to wear and delamination [120]. The coating delamination would induce implant loosening, while the wear debris of metal particles could lead to chronic inflammation. Wear debris have raised concerns about the safety of Ti-PEEK implants. Nevertheless, Ti-PEEK cages have been used for posterior lumbar interbody fusion (PLIF) surgery very recently. Makino et al. employed computed tomography (CT) color mapping to assess bone ongrowth on the Ti-PEEK cages in 24 patients experiencing PLIF. Porous Ti-PEEK cages used were PROSPACE^®^XP (Aesculap AG, Tuttlingen, Germany) [121]. A total of 248 surfaces of cage frames were assessed from the patients at six months after surgery. Bone ongrowth was observed in 134 of 248 images of interbody cages (54.0%) by CT color mapping [121]. 

#### 2.1.3. TiO_2_-Coated PEEK Implants

Titanium dioxide (titania; TiO_2_) exhibits good bioactivity and photocatalytic antibacterial properties [122,123,124,125]. Titania has been deposited on stainless steel pins of fixation devices, showing the ability to reduce the pin site infection [126]. So TiO_2_ layer can be deposited on PEEK for enhancing its osteogenic and antibacterial activities. Titania exists three polymorphs including anatase, rutile and brookite [125]. Titania can react readily with water molecules in aqueous environments, forming hydroxyl groups on its surface [127]. The hydroxyl groups of TiO_2_ provide active sites for depositing bone-like calcium phosphate (apatite) in a simulated body fluid (SBF). The calcium and phosphate ions can bind to the hydroxylated TiO_2_ surface, facilitating osseointegration accordingly [128]. 

**Figure 9:**
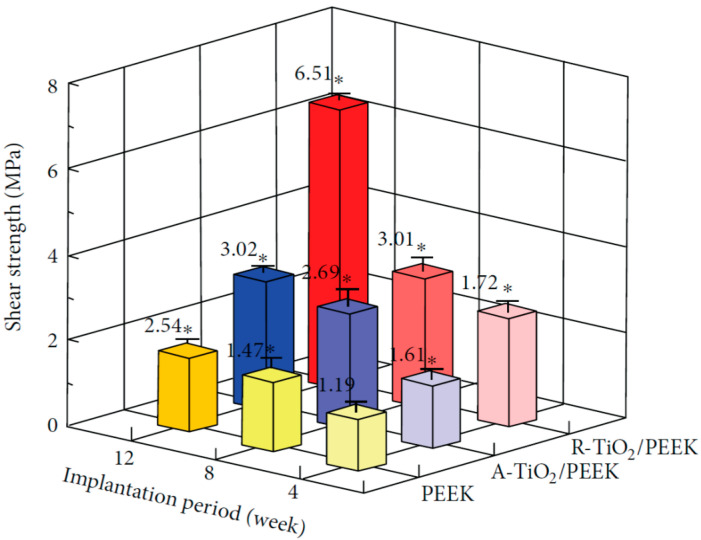
Shear strength between the bone tissue and implant for pure PEEK, A-TiO_2_/PEEK and R-TiO_2_/PEEK at different implantation periods. * *p* < 0.05 compared to PEEK at 4 weeks after implantation. Reproduced from [129] with permission of Hindawi under the Creative Commons Attribution license.

Recently, Tsou et al. deposited TiO_2_ layer on PEEK using arc ion plating (AIP) for spinal implant applications. Anatase-TiO_2_ (A-TiO_2_) and rutile-TiO_2_ (R-TiO_2_) coatings were achieved by controlling the target current and substrate bias voltage during deposition. The coated samples were implanted into the femurs of New Zealand white male rabbits. The shear stress needed for disrupting the bone-implant interface at different time points was determined by the push-out test [129]. The R-TiO_2_/PEEK implant exhibits a shear strength of 6.51 MPa at 12 weeks of implantation, being 2.5 times higher than the shear stress of pure PEEK (Figure 9). The R-TiO_2_/PEEK implant shows superior osseointegration and fixation than A-TiO_2_/PEEK due to the abundance of negatively charged hydroxyl groups on its surface [129]. Shimizu et al. employed the sol-gel technique to deposit a thin TiO_2_ layer on PEEK [99,130]. The sol-gel method is advantageous over plasma spraying and AIP due to its low processing temperature, thus without damaging the structure of PEEK. They demonstrated that TiO_2_-PEEK surfaces favor the formation of apatite upon soaking in SBF. Those surface modified PEEK implants exhibit better bone-bonding properties than uncoated PEEK on the basis of in vivo biomechanical and histological examinations of rabbit tibia model [130]. Despite these benefits, TiO_2_-PEEK implants are not readily available for clinical applications.

## 3. PEEK-Based Microcomposites

### 3.1. PEEK/HA Microcomposites

**Figure 10:**
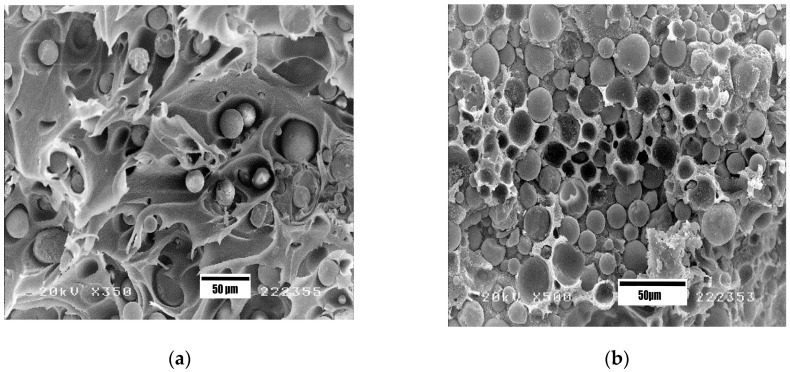
SEM fractographs of (**a**) PEEK-10 vol% mHA and (**b**) PEEK-40 vol% mHA microcomposites, showing debonding of mHA particles from the PEEK matrix. Reproduced from [131] with permission of Elsevier.

The mechanical strength and stiffness of polymers can be enhanced by adding reinforcing materials of micro/nano scale, forming the so-called “microcomposites” and “nanocomposites”. As mentioned, the modulus of elasticity of pure PEEK is 3.7–4.0 GPa [27,28], being lower than that of human cortical bone ranging from 7 to 30 GPa [9]. Therefore, many efforts have been spent by the researchers in enhancing the stiffness and strength of PEEK by reinforcing with HA microparticles, HA whiskers and carbon fibers for load-bearing orthopedic applications [39,40,41,42,43,44,45,46,47,131,132,133,134,135,136,137,138]. The elastic modulus of HA is 120.6 GPa [26], so it can be used as a reinforcement to stiffen PEEK. Abu Bakar et al. incorporated 5–40 vol% mHA into PEEK, resulting in microcomposites with improved stiffness [39,131]. However, the tensile strength of PEEK-mHA microcomposites was smaller than that of pure PEEK. The tensile strength of PEEK decreased markedly from 78.30 MPa to 64.71 and 43.76 MPa by adding 10 vol% and 40 vol% mHA, respectively. The reduction in tensile strength of PEEK-mHA microcomposites was attributed to a poor mHA-PEEK interfacial bonding, leading to decohesion of mHA particles from the polymer matrix. Moreover, the inclusion of 40 vol% mHA into a ductile PEEK matrix led to brittle fracture (Figure 10a,b). The final failure strain of PEEK reduced markedly from 28.93% to 19.23% and 0.98%, respectively by adding 10 vol% and 40 vol% mHA.

**Figure 11:**
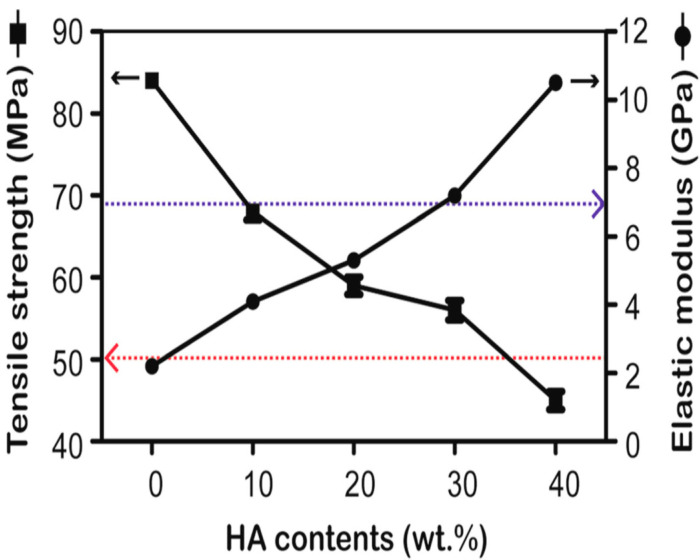
Tensile strength and elastic modulus vs filler content of the PEEK-mHA composites. The blue dotted line denotes low modulus limit of cortical bone (7 GPa). The red dotted line indicates the low tensile strength range of cortical bone (50 MPa). Reproduced from [135] with permission of BioMed Central under the terms of the Creative Commons Attribution 4.0 International license.

**Figure 12:**
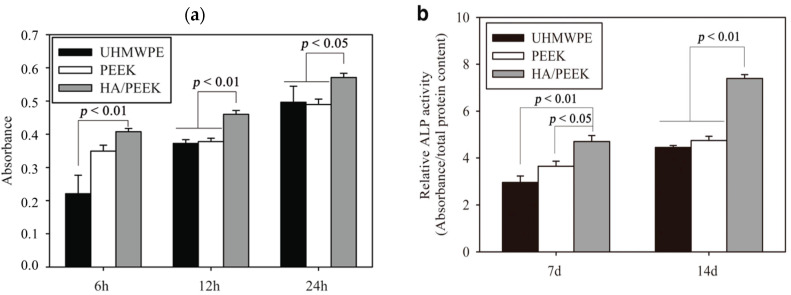
(**a**) Attachment of murine pre-osteoblastic cells (MC3T3-E1) and (**b**) ALP activity of osteoblasts cultured on different material surfaces for different time points. Reproduced from [135] with permission of BioMed Central under the terms of the Creative Commons Attribution 4.0 International license.

**Figure 13:**
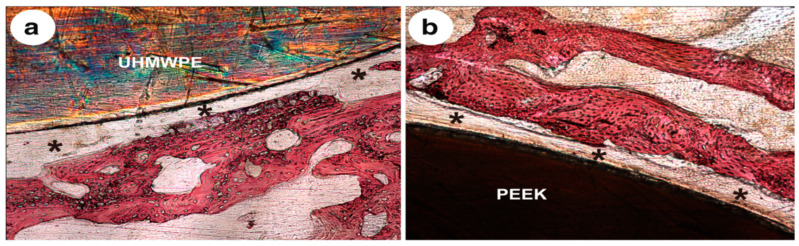

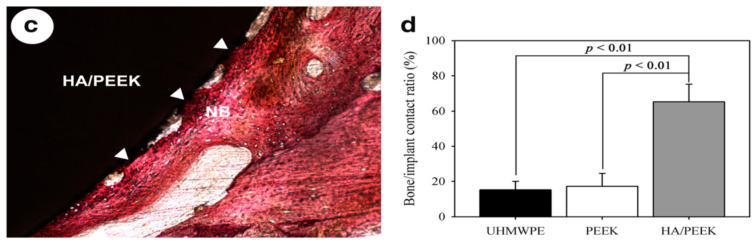
Histological observation after 8 weeks of implantation into rabbit cranial defects: (**a**) UHMWPE, (**b**) PEEK, (**c**) PEEK/30 wt% mHA, and (**d**) quantitative analysis of bone/implant contact ratio. NB: new bone. Black asterisks indicate the fibrous connective tissue, and white arrows denote the bone contact. Reproduced from [135] with permission of BioMed Central under the Creative Commons Attribution 4.0 International license.

Very recently, Ma and Guo injection molded PEEK microcomposites filled with 10, 20, 30, and 40 wt% mHA. The composite samples were cultured with MC3T3-E1 cells, and then implanted into rabbit cranial defects [135]. Figure 11 shows the tensile strength and modulus of elasticity of composites as a function of mHA content. This figure reveals an increase of stiffness but a decline in tensile strength of microcomposites with increasing mHA content. The PEEK/30 wt% mHA and PEEK/40 wt% mHA composites show higher elastic modulus than the low modulus range of cortical bone (7 GPa). However, the tensile strength of PEEK-40wt% mHA composite is smaller than the low tensile strength range of cortical bone, i.e., 50 MPa [9]. So only PEEK/30 wt% mHA composite exhibits tensile properties close to cortical bone. This figure also reveals that that tensile strength of pure PEEK is 84 MPa, almost one-half of the upper limit of tensile strength of cortical bone (150 MPa) [9]. This implies that pure PEEK can sustain the applied load. In the composite materials, polymeric matrix transfers the applied stress to the fillers for load-bearing capacity during tensile testing. Because of poor mHA-PEEK interfacial bonding, inefficient stress transfer between the matrix and mHA is expected. As a result, the PEEK matrix solely carries the load. However, the volume content of PEEK matrix decreases markedly by adding 30 wt% and 40 wt% mHA. This leads to a sharp decline in the tensile strength of PEEK/30 wt% mHA and PEEK/40 wt% mHA composites. As recognized, the interfacial bonding of ceramic fillers and the polymer matrix can be improved by adding coupling agents like silanes. However, silane agents are known to cause irritation of the eyes, skin and respiratory tract. Therefore, the use of coupling agents in biomedical implants must be avoided. Figure 12a,b show the cell attachment and ALP activity of murine pre-osteoblastic cell line (MC3T3-E1) on pure PEEK, UHMWPE, and PEEK/30 wt% mHA composite at different time points. Apparently, the composite exhibits better cell adhesion and higher ALP activity level than pure PEEK and UHMWPE. Figure 13a–c show the respective histological examination of pure PEEK, UHMWPE, and PEEK/30 wt% mHA composite after 8 weeks of implantation. A layer of fibrous connective tissue can be readily seen in UHMWPE and PEEK (Figure 13a,b). In contrast, new bone tissues are formed and contacted closely with the PEEK-30wt% mHA composite (Figure 13c). Furthermore, the composite exhibits higher bone/implant contact ratio than UHMWPE and PEEK (Figure 13d).

### 3.2. PEEK/Carbon Fiber Microcomposites

PEEK-carbon fiber (CF) composites have been explored for orthopedic applications in posterior lumbar interbody fusion implants, screws, intramedullary nails, acetabular cups and dental implants [43,44,45,46,47,136,137,138,139,140,141,142,143]. Pure PEEK is inadequate for those applications because of insufficient stiffness. In particular, PEEK/CF composites are useful for load-bearing applications such as femoral stems of hip joint prostheses and bone fracture plates [144,145,146,147,148]. PEEK/CF materials offer the possibility of altering the stiffness of implants to match the modulus of host bone by changing the volume fraction, length and orientation of reinforcing carbon fibers. As such, the elastic modulus of PEEK-CF composites can be tailored readily to reach the stiffness of cortical bone. The precursors generally used to manufacture commercial CFs include polyacrylonitrile (PAN) and pitch. PAN-based CFs can be classified into high strength fibers with a stiffness of 230–300 GPa and tensile strength of 3500–6400 MPa, and high modulus fibers with a stiffness of 370–590 GPa and tensile strength of 4000–5500 MPa. Pitch-based CFs prepared from petroleum products generally have larger diameter and lower strength than PAN-based CFs. The stiffness of pitch-based CFs varies from 50 to 900 GPa [146]. 

Endolign^®^ composites consist of PEEK matrix and continuous CFs (65 vol%) in the form of a pre-impregnated tape have been developed by Invibio Ltd. (Lancashire, UK) in 2006. In those composites, carbon fibers in the form of pre-preg tapes are arranged into unidirectional (U.D.) or multidirectional (M.D.) configurations. U. D. Endolign^®^ with aligned fibers in the loading direction has a stiffness of 150 GPa and tensile strength of 2000 MPa, while multidirectional product shows a stiffness of 70 GPa and tensile strength of 900 MPa. Continuous carbon fiber preforms in M.D. Endolign^®^ are arranged in 0°/90°/45° orientations relative to the tensile axis [146]. Thus M.D. Endolign^®^ composite with a stiffness closer to cortical bone is more suitable for fabricating load-bearing implants. PEEK and preforms with high CF content and long fiber length cannot be processed in conventional injection molders to produce microcomposites. Continuous CFs reinforced PEEK composites are generally manufactured using compression molding, autoclave molding, filament winding, and pultrusion. Such high-temperature polymer composite processing techniques are typically developed for aerospace and automotive industries for forming components with complex geometries [149].

The mechanical properties of PEEK composites reinforced with continuous CFs are anisotropic. Longitudinal tensile modulus and strength of unidirectional fiber-reinforced composite in which fibers align parallel to the loading direction are considerably higher than transverse tensile modulus and strength (fibers normal to the tensile loading direction). In contrast, the mechanical properties of PEEK composites reinforced with randomly oriented short carbon fibers (SCFs) are isotropic. SCFs are much cheaper than continuous CFs, and PEEK/SCF composite can be readily fabricated using conventional extrusion and injection molding techniques. The PEEK/30 wt% SCF composite exhibits a stiffness of 24 GPa and tensile strength of 214 MPa [42]. Very recently, Bonnheim et al. examined tensile and fatigue properties of orthopedic grade PEEK reinforced with 30 wt% PAN- and 30 wt% pitch-based SCFs. The stiffness of PAN- and pitch-based fibers was 540 GPa and 280 GPa, respectively [150]. The elastic modulus, tensile strength, and fracture strain of injection molded PEEK/30 wt% SCF (PAN) are 18.5 ± 2.3 GPa, 192 ± 17 MPa and 1.9 ± 0.2%, while those of PEEK/30 wt%SCF (pitch) are 12.5 ± 1.3 GPa, 145 ± 9 MPa and 2.2 ± 0.2%, respectively. Rupp and coworkers employed FFF method to print PEEK/5 wt% SCF with a stiffness of 7.37 ± 1.22 GPa, thus reaching the low modulus range of cortical bone [45]. The close resemblance of the mechanical properties of PEEK/SCF composites with human cortical bone rendering them a potential biomaterial for orthopedic applications. Table 1 summarizes the tensile properties of PEEK-based microcomposites.

#### PEEK/CF Hip Prostheses

In general, little work has been reported on the use of PEEK/CF composites for load-bearing hip stem applications. Furthermore, there have been concerns relating the inertness of PEEK/CF composites, which prevents the formation of bone tissues around the implants. Earlier study conducted by Scotchford et al. showed that the attachment and proliferation of primary human osteoblasts on PEEK/CF disks were not significantly different to those on Ti-6Al-4V alloy [151]. However, the in vitro cell culture studies were conducted for short time periods, and the results could not directly reflect the safe use of composite implants in the clinical sector.

Nakahara et al. implanted HA-coated PEEK/65vol% CF (unidirectional) composite hip stem in an adult ovine model for 12 months. The HA-coated PEEK/CF hip stem exhibited bone on-growth fixation and minimal stress shielding. However, bone resorption and osteopenia were observed in HA-coated Ti-6Al-4V alloy hip stem [147]. In another study, they also conducted in vivo implant fixation of cementless and cemented PEEK/CF hip stems in an ovine model over 52 weeks of implantation. Uncemented HA-coated PEEK/CF stem showed bony ongrowth fixation. Cemented PEEK/CF stem without HA coating provided a stable long-term fixation showing no gaps at both the bone-cement and cement-stem interfaces [148]. Nakahara et al. also implanted HA-coated PEEK/CF into the femurs of rabbits. The interfacial shear strength of HA-coated PEEK/CF and uncoated PEEK/CF implants was determined using the pull-out test [152]. After implantation for 6 weeks, the shear strength of HA-coated PEEK/CF implant (15.7  ±  4.5 MPa) was twice the strength of uncoated PEEK/CF implant (7.7  ±  1.8 MPa). At 12 weeks, the shear strength of HA-coated PEEK/CF and pristine PEEK/CF implants was found to be 17.4  ±  3.6 MPa and 8.3  ±  3.0 MPa, respectively. These results revealed the effectiveness of HA in enhancing the bone growth around the coated implant. Similarly, Suska et al. also indicated that plasma-sprayed HA-PEEK/CF implants inserted into the femurs and tibias of rabbits showing better osseointegration than uncoated PEEK/CF implants [153]. In a recent study of Stubinger et al., HA-coated PEEK/CF implants inserted into the pelvis of sheep showed a significant improvement of osseointegration over uncoated PEEK/CF samples after 2 and 12 weeks of implantation [154]. 

UHMWPE has been widely used as a bearing surface material of hip prostheses articulating with a metallic or ceramic counterface. The wear debris released by UHMWPE acetabular cup into the surrounding tissue often leads to inflammation, causing osteolysis and implant failure [155]. PEEK exhibits a three times higher friction coefficient than UHMWPE [156,157], rendering PEEK with poorer wear performance than UHMWPE. Taking advantages of high stiffness and strength, PEEK/SCF composite exhibits a higher wear resistance than pure PEEK [158,159]. From the hip simulator testing for 10 million cycles, the wear rate of PEEK/30 wt% SCF (pitch) composite articulating against a zirconia ceramic head is almost two orders of magnitude lower than that of UHMWPE [160]. Thus PEEK/SCF composites can provide good stability for acetabular cup in hip prostheses sliding against hard femoral head. However, PEEK/30 wt% SCF (pitch) and PEEK/30 wt% SCF (PAN) composites exhibit higher wear rates than UHMWPE using a multidirectional cylinder-on-flat tribometer with bovine serum as lubricant. The multidirectional wear test simulates high contact stress conditions at the knee [161]. 

The wear resistance of polymeric materials is dependent on the applied stress, sliding distance, temperature, the presence of lubricant, etc. At a high applied stress or contact pressure, the wear rate increases markedly as expected. This is due to the removal of a large fraction of volume of material as wear debris. Recently, Brockett et al. studied the wear performance of PEEK and PEEK/30 wt% SCF (pitch) composite articulating with a clinically CoCrMo femoral bearing in a low conformity knee replacement using a six-station multidirectional pin-on-plate wear simulator. They demonstrated that PEEK and PEEK/30 wt% SCF composite exhibit very high wear rates under a low-conforming, high contact stress knee condition. So, they are unsuitable to replace UHMWPE in total knee replacement [139]. 

## 4. PEEK-Based Nanocomposites

### 4.1. PEEK-nHA Nanocomposites

**Figure 14:**
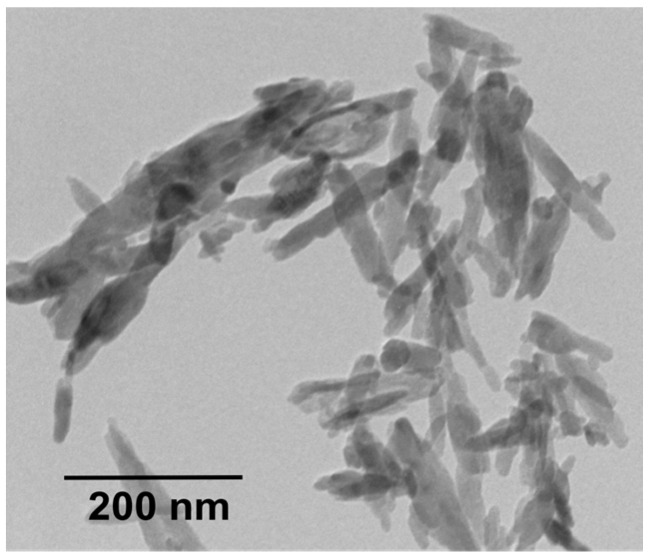
Transmission electron micrograph of nHA rods. Reproduced from [63] with permission of MDPI.

As mentioned, human bones consists of 70% nHA platelets and 30% collagen fibrils. Thus, bionanocomposites for hard tissue regeneration are developed and designed by combining PEEK and nHA with nanometer dimension [60,62,63,87,162,163,164]. Nanohydroxyapatite can be prepared by means of co-precipitation, sol-gel, and hydrothermal techniques [165]. It generally exhibits a rod-like feature (Figure 14). Synthetic nHA has been reported to promote the adhesion, proliferation and differentiation of osteoblasts, and new bone formation [54,55,56,57,58,59]. Furthermore, nHA favors more protein adsorption than mHA, leading to an initial cell attachment on its surface [166]. 

**Figure 15:**
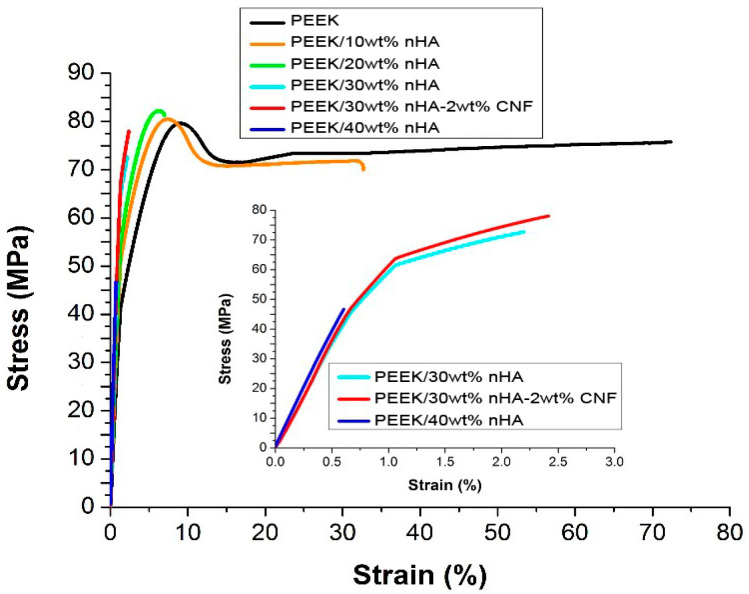
Tensile stress-strain curves of PEEK/nHA nanocomposites prepared by melt-compounding followed by injection molding. Reproduced from [62] with permission of Royal Society of Chemistry.

Ma and coworkers mixed PEEK powders and nHA particles in a ball mill followed by injection molding [87,162]. The stiffness of PEEK, PEEK/20 wt% nHA and PEEK/40wt% is reported to be 2.20 ± 0.17 GPa, 3.40 ± 0.20 GPa and 4.60 ± 0.12 GPa, respectively. The stiffness of ball-milled/injection molded PEEK is smaller than the modulus of PEEK reported in the literature, i.e., 3.8 GPa [27]. The tensile strength of PEEK, PEEK/20 wt% nHA and PEEK/40 wt% is 84.0 ± 1.9 MPa, 81.0 ± 2.4 MPa and 75.0±2.7 MPa, respectively. Apparently, there exists no reinforcing effect of nHA for PEEK. This issue results from the mixing of PEEK powders and nHA for forming composites. Commercial PEEK powders are inadequate for injection molding purposes. On the contrary, Tjong and coworkers used injection molded grade PEEK pellets (PEEK-Optima, Invibio) as the matrix material of nanocomposites. The PEEK pellets and nHA were melt-mixed in a Brabender compounder. The extruded strands were chopped into pieces by a pelletizer and loaded into Brabender again to achieve homogeneous filler dispersion in the polymer matrix. The compounded pellets were finally injection molded to produce composite samples. The PEEK/nHA nanocomposites showed a slight improvement in tensile strength by adding 10 wt% nHA and 20 wt% nHA at the expense of tensile ductility. Thereafter, it decreased with increasing nHA content, i.e., ≥ 30 wt% nHA (Figure 15) [62]. 

**Figure 16:**
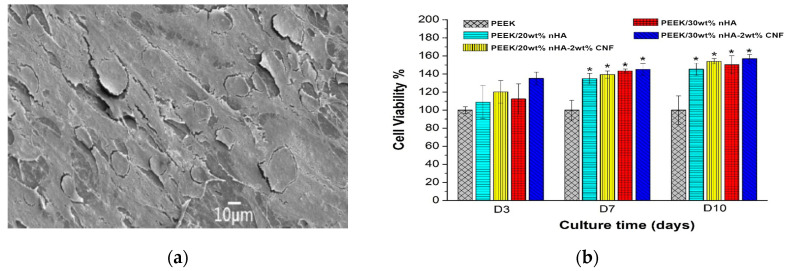
(**a**) Scanning electron micrograph showing the attachment of Saos-2 cells on PEEK/15 vol% nHA after culturing for 3 days. (**b**) Cell viability of Saos-2 cells on pure PEEK and its nanocomposites at different time points. * represents *p* < 0.05. Reproduced from [62] with permission of Royal Society of Chemistry.

The elastic modulus of PEEK is 3.87 ± 0.10 GPa and increases to 4.34 ± 0.08 GPa and 4.92 ± 0.06 GPa by adding 10 wt% and 20 wt% nHA, respectively. The stiffness of PEEK/40 wt% nHA reaches 7.85 ± 0.11GPa, being higher than the lower modulus range of cortical bone. However, PEEK/40wt% nHA with a tensile ductility of 0.69 ± 0.21% is very brittle; this value is smaller than the fracture strain of cortical bone ranging from 1–3%. So, this stiff and brittle composite is unsuitable for bone replacement. The stiffness of PEEK/30wt% nHA is 6.20 ± 0.13 GPa, while the tensile strength (70.56 ± 3.22 MPa) and fracture ductility (2.71%) of this nanocomposite meet the tensile properties of cortical bone. The elastic modulus of PEEK/30 wt% nHA can be further improved to 6.54 GPa by adding 2wt% carbon nanofibers (CNFs). The incorporation of 30 wt% nHA into PEEK promotes the adhesion of human osteoblastic cells (Saos-2) greatly (Figure 16a). Comparing with pure PEEK, bone cell viability is dramatically improves by adding 20 wt% and 30 wt% nHA to PEEK as revealed by 2-(4-iodophenyl)-3-(4-nitrophenyl)-5-(2,4-disulfophenyl)-2H-tetrazolium (WST-1) assay (Figure 16b). 

**Figure 17:**
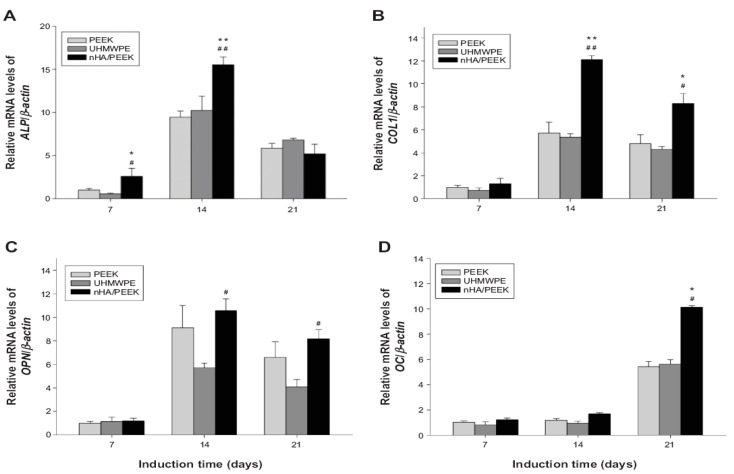
Differentiation of MC3T3-E1 cells on PEEK, UHMWPE and PEEK/40wt% nHA samples determined by real-time polymerase chain reaction: (**a**) ALP, (**b**) COL1, (**c**) OPN, and (**d**) OC gene expressions. * Significant difference compared with PEEK (*p* < 0.05); ** significant difference compared with PEEK (*p* < 0.01); # significant difference compared with UHMWPE (*p* < 0.05); ## significant difference compared with UHMWPE (*p* < 0.01). Reproduced from [87] with permission of Dove Press under the Creative Commons Attribution license.

Ma et al. studied in vitro cell adhesion, proliferation and differentiation of murine MC3T3-E1 pre-osteoblasts on PEEK/40 wt% nHA despite its poor stiffness (4.60 ± 0.12 GPa), i.e., smaller than the low modulus range of cortical bone [87]. Comparing with pure PEEK and UHMWPE, PEEK/40 wt% nHA nanocomposite favors the adhesion and proliferation of MC3T3-E1 cells. Figure 17a–d show the real time polymerase chain reaction (RT-PCR) analysis of osteogenic marker genes of MC3T3-E1 cells cultured on PEEK, UHMWPE and PEEK/40 wt% nHA samples for different time points. PEEK/40 wt% nHA induces higher expression levels of osteogenic differentiation-related genes. In general, the differentiation of pre-osteoblasts occurs via three phases, i.e., proliferation, maturation of matrix, and mineralization [167]. During the proliferation phase, osteoblasts secrete bone matrix proteins including collagen type 1 (COL1), osteopontin (OPN) and fibronectin. Subsequently, they mature the extracellular matrix (ECM) with ALP and collagen. The final phase involves the mineralization of bone by expressing osteocalcin (OC), facilitating calcium phosphate deposition [168]. From Figure 17a, a higher expression of ALP is observed on PEEK/40 wt% nHA compared to PEEK and UHMWPE at 7 and 14 days. The ALP activity of all samples then decreases at day 21 as expected, because ALP is an early marker of differentiation of pre-osteoblasts. Furthermore, bioactive nHA fillers are effective to promote the differentiation of osteoblasts. As such, PEEK/40 wt% nHA nanocomposite shows a higher COL1 and OPN expressions than pure PEEK and UHMWPE (Figure 17b,c). Finally, PEEK/40 wt% nHA nanocomposite exhibits the highest OC expression level at day 21 associated with the mineralization of matrix (Figure 17d). Recently, Zhao et al. also reported that melt-mixed/injection molded PEEK/40 wt% nHA composite exhibits enhanced adhesion and proliferation of human osteoblast-like MG-63 cells than pure PEEK [169]. 

**Figure 18:**
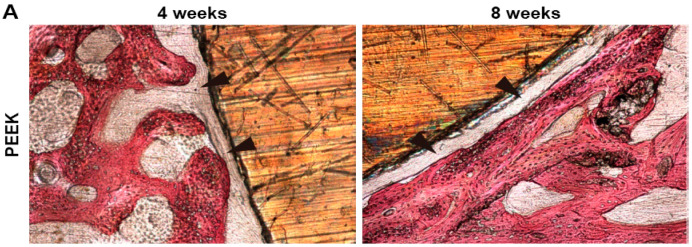

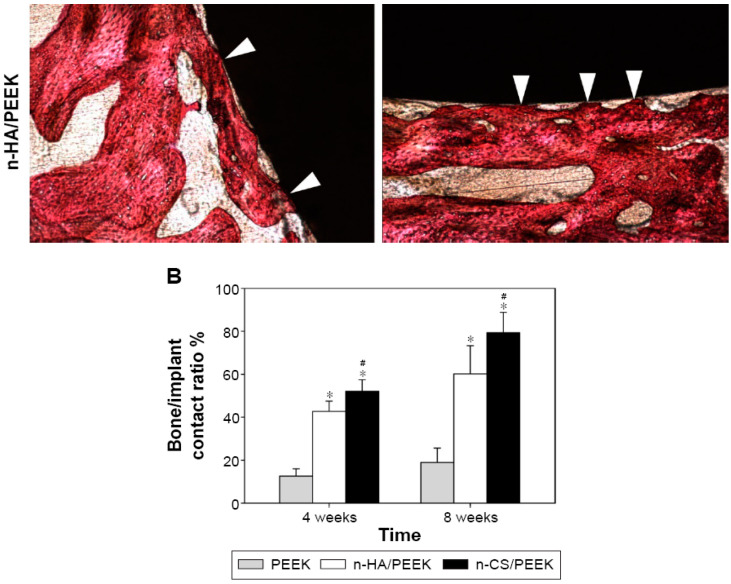
Histological observation of osseointegration of pure PEEK and PEEK/40wt% nHA samples after 4 and 8 weeks implantation into full-thickness cranial defects of rabbits. (**A**) Histological images. The black arrows indicate fibrous connective tissue, and white arrows indicate bone contact. (**B**) Comparison of percentage of bone/implant contact among PEEK, PEEK/40 wt% nHA and PEEK/40 wt% *n*-CS implants. ∗ denotes a significant difference compared with PEEK (*p* < 0.01), and # indicates a difference compared with PEEK/40wt% nHA (*p* < 0.05). Reproduced from [163] with permission of Dove Press under Creative Commons Attribution license.

Ma et al. also implanted PEEK and PEEK/40wt% nHA into full-thickness cranial defects of rabbits for 4 and 8 weeks. For comparison, injection molded PEEK/40 wt% calcium silicate (*n*-CS) was also implanted into the defect model [163]. Figure 18a shows histological micrographs of PEEK and PEEK/40 wt% nHA samples after 4 and 8 weeks of implantation. There exists no direct bone contact for pure PEEK at 4 and 8 weeks. However, a layer of fibrous connective tissue as indicated by the black arrows can be readily seen. The fibrous tissue are commonly observed when foreign materials are implanted into a mammalian body. In contrast, bone tissues are formed on the PEEK/40 wt% nHA composite and contact directly with the implant surface as indicated by white arrows. The direct contact between the bone and implant, and the lack of fibrous connective tissue, demonstrating good osseointegration of the implant. Figure 18b reveals that both the PEEK/40 wt% nHA and PEEK/40 wt% *n*-CS implants exhibit higher bone contact than pure PEEK, especially for PEEK/40wt% *n*-CS. 

**Figure 19:**
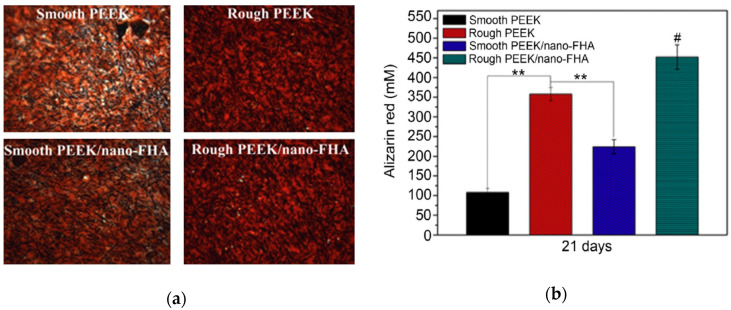
(**a**) The formation of mineralized extracellular matrix of hMSCs cultivated on different PEEK-based samples after staining with Alizarin Red S at day 21. Alizarin red dye stained the cells containing calcium deposits in dark red color. (**b**) Mineralization of hMSCs seeded on both smooth and rough PEEK as well as its composite groups at day 21 using Alizarin Red S assay. ** represents *p* < 0.01, and # represents *p* < 0.05 compared with other groups. Reproduced from [164] with permission of Elsevier.

**Figure 20:**
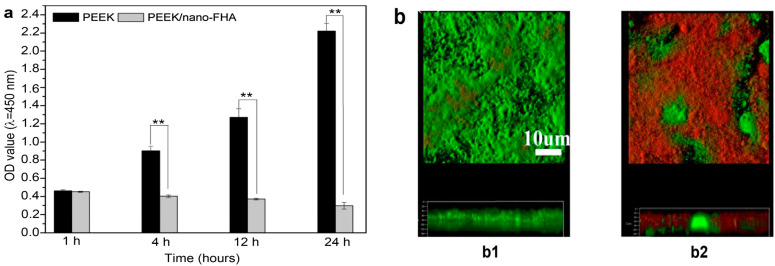
(**a**) Bactericidal activities of PEEK and PEEK/nano-FHA composite samples against *S. mutans* cultured for different time points. (**b**) Live/dead cell staining of *S. mutans* cultured on PEEK (**b1**) and PEEK/nano-FHA (**b2**) for 14 days. ** represents *p* < 0.01. Reproduced from [164] with permission of Elsevier.

Apart from osseointegration, the resistance to bacterial infection is another factor influencing the clinical success of implants. In particular, the failure of dental implants is associated with bacterial colonization and biofilm formation, leading to the inflammation and deterioration of the tissue implant integration [170]. *Streptococcus mutans* is a bacterial species residing in the human oral cavity, forming biofilm on dental implants and causing peri-implant diseases [171]. Recently, Wang et al. fabricated PEEK composite filled with 40 wt% nano-fluorohydroxyapatite (nano-FHA) using compression molding process. Fluorine was incorporated into nHA to prevent bacterial colonization [164]. Some PEEK and PEEK/nano-FHA samples were blasted with TiO_2_ particles for surface roughening. The PEEK/nano-FHA composite promoted the adhesion, proliferation and differentiation of human MG63 osteoblasts as expected. Moreover, hMSCs were also seeded on these samples. The hMSC cells seeded on rough PEEK and PEEK/nano-FHA surfaces showed more brighter red staining than smooth sample groups at 21 days as revealed by Alizarin Red S staining, demonstrating mineralization of hMSCs at the final differentiation stage (Figure 19a,b). Alizarin red agent stained the cells containing calcium deposits in dark red color. Figure 20a,b showed the growth of *S. mutans* and biofilm formation on rough PEEK and PEEK/nano-FHA as determined by bacteria live/dead assay. Dead bacteria were fluoresced red and living cells with intact cell membranes were labeled green. The results were analyzed with spectrophotometry and confocal laser scanning microscopy (CLSM). In Figure 20a, few bacteria were attached on pure PEEK and composite samples at 1h. However, bacterial colonies on PEEK increased sharply with time up 24 h, while low bacterial densities developed on the PEEK/nano-FHA, so forming a surface resistant to bacterial adhesion. The CLSM image of PEEK revealed the formation of biofilm with intense green fluorescence on its surface at prolonged culture period of 14 days. In contrast, numerous dead bacterial cells with red fluorescence were seen on the PEEK/nano-FHA surface (Figure 20b). From in vivo beagle dog model, rough PEEK/nano-FHA implant showed good bioactivity, osseointegration, and bone-implant contact. 

### 4.2. PEEK-Metal Oxide Nanocomposites

Metal oxides such as titania and zinc oxide (ZnO) are wide-bandgap semiconductors having photocatalytic properties. Excitation those oxides with photons of energy greater than the band-gap, an electron in the valence band (VB) is ejected and jumped to the conduction band, producing a positive hole in the VB. As the photogenerated electron-hole pairs arrive the semiconductor surface, they react with surface-adsorbed water and oxygen to generate reactive oxygen species (ROS) like hydroxyl (•OH), superoxide anion (O^2−^), and hydrogen peroxide (H_2_O_2_). [125,172,173]. Photogenerated ROS can inactivate microbial pathogens, rendering TiO_2_ and ZnO effective nanomaterials for bacterial killing [174,175]. Because of the wide-bandgap, ROS can be induced on TiO_2_ under ultraviolet (UV) light irradiation. This implies that visible light with long wavelengths is incapable of generating ROS on TiO_2_ for bacterial inactivation. UV radiation is known to be harmful to humans, especially for the skin and eyes. On the contrary, ZnO can generate ROS under visible light and even in the dark due to the presence of surface vacancy defects [176]. Moreover, ZnO can release Zn^2+^ ions upon direct contact with bacteria strains, thereby damaging their cell membranes [177]. Thus, ZnO nanoparticles are potential fillers for fabricating antibacterial PEEK-based nanocomposites for orthopedic applications. Surgical site infections due to bacterial contamination of medical implants can lead to a life-threatening postoperative complication [178]. The bacterial infections have motivated the researchers to develop medical implants with bactericidal activity.

**Figure 21:**
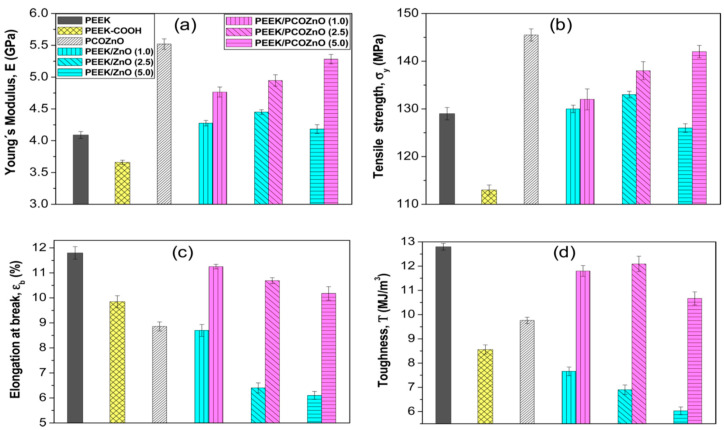
Tensile properties of PEEK/ZnO and modified PEEK/ZnO nanocomposites: (**a**) elastic modulus, (**b**) tensile strength, (**c**) strain at break, and (**d**) toughness. The values in parentheses of (**a**) indicate the weight percentage of ZnO. PCOZnO: PEEK−CO−O−CH2−ZnO. Reproduced from [179] with permission of American Chemical Society.

**Figure 22:**
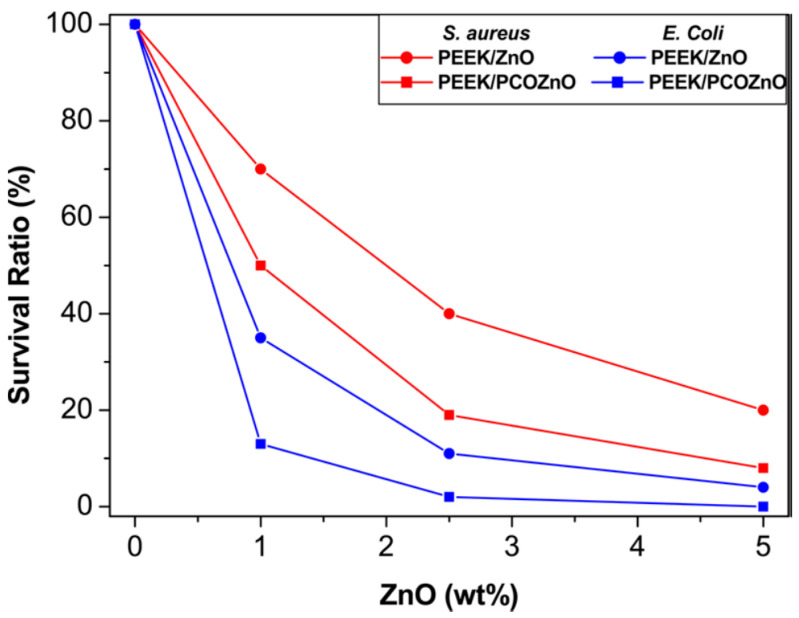
Antibacterial activity of PEEK/ZnO and modified PEEK/ZnO nanocomposites as a function of ZnO content. Reproduced from [179] with permission of American Chemical Society.

Díez-Pascual and Díez-Vicente fabricated PEEK/ZnO and silane-modified PEEK/ZnO nanocomposites using melt-mixing process. To improve interfacial filler-matrix bonding, ZnO nanoparticles (NPs) were surface modified with 5,6-epoxyhexyltriethoxysilane (EHTES), methanol and hydrochloric acid to yield ZnO−CH_2_OH. Meanwhile, PEEK was treated with sodium borohydride (NaBH_4_) and dimethyl sulfoxide to form PEEK−COOH [179]. The PEEK−COOH was grafted to the surface of ZnO−CH_2_OH to create PEEK−CO−O−CH_2_−ZnO, terming as PCOZnO. The PCOZnO of desired contents were melt-mixed with PEEK to form modified PEEK nanocomposites. EHTES is toxic to humans by irritating the skin, eyes and respiratory tract system. NaBH_4_ is explosive and toxic to human cells. The use of those chemical regents should be restricted for fabricating human implants. Figure 21a–d show the tensile properties of PEEK/ZnO and modified PEEK/ZnO nanocomposites. The stiffness and tensile strength of PEEK/ZnO nanocomposites improve slightly by adding 1.0 and 2.5 wt% ZnO NPs. At 5.0 wt% ZnO, the composite exhibits a decline in both the tensile modulus and strength due to the agglomeration of ZnO NPs. However, elastic modulus and tensile strength of modified PEEK/ZnO nanocomposites increase with increasing filler content at the expense of tensile elongation and toughness. The improvement in tensile strength and stiffness are attributed to enhanced interfacial filler-matrix bonding. Figure 22 shows the antibacterial activity of PEEK/ZnO and modified PEEK/ZnO nanocomposites against Gram-positive *Staphylococcus aureus* and Gram-negative *Escherichia coli*. As recognized, *S. aureus* often induces implant infections, which are hard to treat due to its antibiotic resistance and ability to form biofilm [180]. Pure PEEK exhibits no bactericidal activity as expected. The survival ratio of both bacterial strains decreases with increasing nanofiller content, especially at filler contents ≥ 2.5 wt%. PEEK/ZnO and modified PEEK/ZnO nanocomposites exhibit higher antibacterial efficacy against *E. coli* than *S. aureus*. This is due to a difference in the cell wall structures of both bacterial strains [181]. The antibacterial efficacy of modified PEEK/ZnO nanocomposites is stronger than PEEK/ZnO counterparts due to their improved filler dispersion. In another study, Díez-Pascual et al. modified ZnO NPs with 3-anilinopropyltrimethoxysilane (APTMS). The silane modified ZnO NPs (mZnO) were mixed with pure PEEK followed by compressing molding. Similarly, the bactericidal activity against *S. aureus* and *E. coli* was enhanced with increasing ZnO or mZnO content [182]. 

### 4.3. PEEK-CNT Nanocomposites

**Figure 23:**
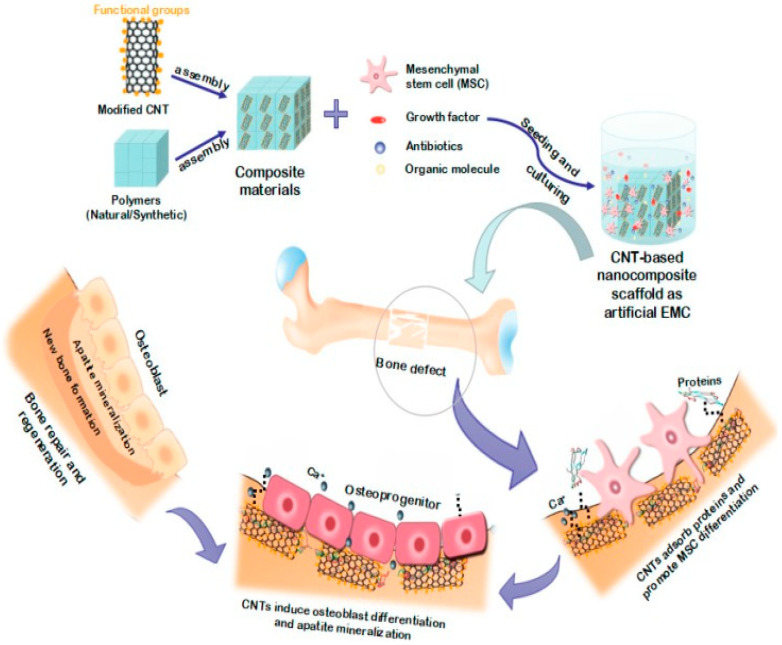
Schematic illustration showing the role of carbon nanotubes for the scaffolding in bone tissue engineering and regeneration. Reproduced from [191] with permission of MDPI under the terms and conditions of the Creative Commons Attribution license.

Carbonaceous nanofilers like graphene and CNTs exhibit extraordinarily high elastic modulus and tensile strength as well as large aspect ratio. Graphene and CNTs generally have an elastic modulus of about 1 TPa [183,184], so they can be used to reinforce polymers at low filler loadings [185,186,187]. Single walled CNT (SWNT) is formed by wrapping a graphene sheet into a cylinder with a diameter of 1–2 nm. Multi walled CNT (MWNT) is generated by rolling multiple graphene sheets into concentric cylinders. SWNTs usually agglomerate into ropes or bundles due to van der Waals forces between carbon atoms. As such, SWNTs are difficult to disperse uniformly as independent nanotubes in the matrix of polymer nanocomposites. In contrast, MWNTs have weaker van der Waals forces, showing less tendency to form ropes and bundles. MWNTs exhibit a very high elastic modulus of 0.9 TPa and tensile strength of 150 GPa [188]. Thus, MWNTs are usually used to reinforce polymers for industrial and biomedical applications. Vapor grown carbon nanofiber (VGCNF) comprises of different arrangement of graphitic layers in herringbone and tubular features, with diameters ranging from 50 to 500 nm [189,190]. The mechanical properties of CNFs are inferior to those of MWNTs due to the presence of more crystalline defects. From biomedical perspective, carbonaceous nanomaterials promote the adhesion, growth and differentiation of bone cells [70,71,72,73,74,75,76,77,78,79,80,81]. For instance, MWNTs show potential applications as structural biomaterials and reinforcing nanofillers for degradable polymer scaffolds for bone tissue engineering (Figure 23) [191,192,193]. Graphene oxide (GO), a graphene derivative, with a Young’s modulus of 207.6 ± 23.4 GPa [194,195], also acting as an effective reinforcement for biodegradable polymer nanocomposites in bone tissue engineering [21,22,192,196]. However, very scarce information is available in the literature relating the mechanical behavior and biocompatibility of PEEK/GO nanocomposites. Very recently, He et al. injection molded PEEK/(0.1–5 wt%)GO nanocomposites using PEEK powders and commercially available GO sheets [197]. The elastic modulus of pure PEEK is around 1.31 GPa, and remains unchanged by adding 0.5 wt% GO. The stiffness increases slightly to 1.34 GPa and 1.38 GPa by adding 1 and 2 wt% GO, respectively. Apparently, the use of raw PEEK powders would have little effect on the stiffness enhancement of PEEK/GO nanocomposites. 

Deng et al. fabricated PEEK/MWNT nanocomposites containing 6.5–15 wt% MWNTs using melt-extrusion and injection molding [198]. The elastic modulus of injection molded PEEK increases from 4.0 GPa to 5.32 GPa, 6.35 GPa and 7.55 GPa by adding 6.5 wt%, 12 wt% and 15 wt% MWNT, respectively. The tensile strength of PEEK increases from 93.55 MPa to 102.15 MPa, 107.14 MPa and 110.90 MPa by adding 6.5 wt%, 12 wt% and 15 wt% MWNT, respectively. The fracture elongation of PEEK is greater than 20% and decreases to 8.28% and 6.28% by adding 12 wt% and 15 wt% MWNT, respectively. Apparently, the tensile characteristics of PEEK/15 wt% MWNTs meet the tensile properties of human cortical bone. The fracture elongation of this nanocomposite can maintain an adequate value of 6.28% due to the crack bridging effect of MWNTs of large aspect ratios, and excellent mechanical flexibility of nanotubes under the application of large deformation strains [199]. Despite the beneficial effect of 15 wt% MWNT addition, the filler content is considered to be relatively high for a nanocomposite, which may induce filler aggregation. 

**Figure 24:**
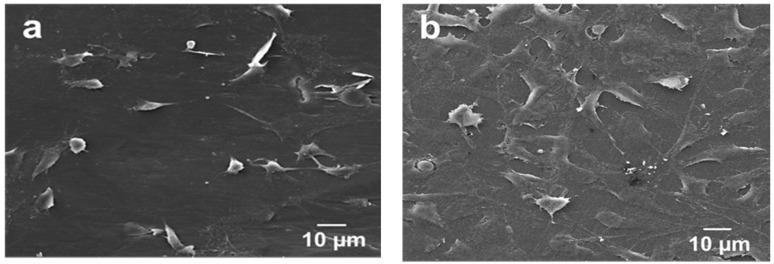
SEM images showing the adhesion of murine MC3T3-E1 pre-osteoblasts on PEEK/3.0 wt% MWNT for (**a**) two and (**b**) four days. Reproduced from [63] with permission of MDPI.

**Figure 25:**
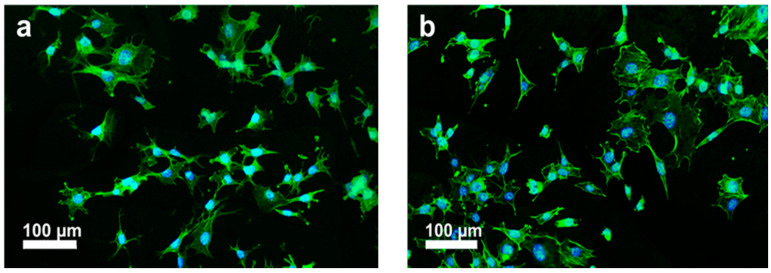
Fluorescence micrographs of murine MC3T3-E1 pre-osteoblasts cultivated on (**a**) PEEK/1.5 wt% MWNT and (**b**) PEEK/3.0 wt% MWNT nanocomposites for 3 days, and stained with F-actin cytoskeleton (green) and nucleus (blue). Reproduced from [63] with permission of MDPI.

Tjong and coworkers injection molded PEEK and its nanocomposites reinforced with 1.5 wt% MWNT and 3.0 wt% MWNT. Low loading levels of MWNTs were added to PEEK to prevent agglomeration of nanotubes [63]. The stiffness, tensile strength and elongation at break of PEEK/1.5 wt% MWNT are 4.21 ± 0.11 GPa, 83.38 ± 0.78 MPa and 57.25 ± 13.20%, respectively. Moreover, the stiffness, tensile strength and elongation at break of PEEK/3.0wt% MWNT are 4.25± 0.85 GPa, 82.08 ± 3.68 MPa, and 56.48 ± 24.90%, respectively. It is evident that the elastic modulus of PEEK/3.0 wt% MWNT nanocomposite is smaller than the low modulus range of cortical bone. However, the addition of 30 wt% nHA to this composite improves its stiffness to 7.13 ± 0.12 GPa, forming the so-called hybrid nanocomposite. Figure 24a,b are the SEM micrographs showing the attachment of murine MC3T3-E1 pre-osteoblasts on PEEK/3.0 wt% MWNT nanocomposite for two and four days, respectively. The adhesion of murine osteoblasts can be clearly seen on PEEK/1.5 wt% MWNT and PEEK/3.0 wt% MWNT composite surfaces by staining F-actin filaments and nuclei of osteoblasts (Figure 25a,b). It is evident that low loading levels of MWNTs promote the adhesion of osteoblasts on bioinert PEEK surface.

## 5. Hybrid Composites

From the above discussion, it appears that the reinforcement made of single type of material is insufficient to achieve desired biocompatibility and mechanical properties of polymer composites for load-bearing applications. The filler reinforcement is the load-carrying component of the composites, while the matrix transfers the applied load to the fillers during mechanical testing. For instance, the elastic modulus, tensile strength, and fracture strain of injection molded PEEK/30 wt% SCF composite meet the tensile property requirement of cortical bone. However, this composite lacks bioactive fillers such as nHA for promoting adhesion, growth and differentiation of osteoblasts on its surface [151]. As aforementioned, the elastic modulus of PEEK/3.0 wt% MWNT is 4.25 ± 0.85 GPa, i.e., smaller than the low modulus range of cortical bone. It can reach 7.13 ± 0.12 GPa by adding an optimal amount of nHA [63]. In this respect, hybridization of fillers of different materials is considered to be effective to obtain desired mechanical stiffness and biocompatibility of resulting biocomposites [200]. 

### 5.1. PEEK-nHA-MWNT Hybrids

**Figure 26:**
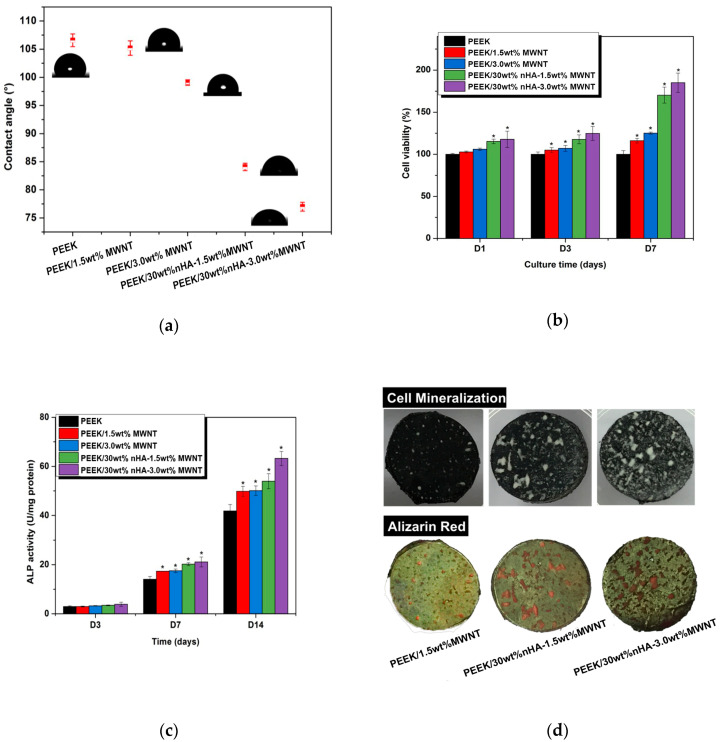
(**a**) Water contact angle values of pure PEEK, PEEK/MWNT nanocomposites and PEEK/nHA-MWNT hybrids. (**b**) WST-1 assay results showing cell viability of murine MC3T3-E1 pre-osteoblasts cultured on PEEK, PEEK/MWNT nanocomposites and PEEK/nHA-MWNT hybrids for 1, 3 and 7 days. * represents *p* < 0.05. (**c**) ALP activity normalized to protein content of MC3T3-E1 cells cultured on PEEK, PEEK/MWNT nanocomposites and PEEK/nHA-MWNT hybrids for different time points. (**d**) Photographs showing calcium mineralization (before staining) on PEEK/1.5 wt%MWNT, PEEK/30 wt%nHA-1.5 wt%MWNT and PEEK/30 wt%nHA-3.0 wt%MWNT specimens cultivated with MC3T3-E1 cells for 28 days (top panel); Calcified nodules on these specimens exhibit dark red appearance after staining with Alizarin red-S (bottom panel). Reproduced from [63] with permission of MDPI under the terms and conditions of the Creative Commons Attribution license.

Tjong and coworkers explored the stiffness enhancement of PEEK/30 wt% nHA by adding low loading levels of CNFs or MWNTs. The results are listed in Table 2 [62,63]. For the purposes of comparison, this Table also summarizes the tensile properties of PEEK-based nanocomposites. By adding 3.0 wt% MWNT to PEEK/30wt% nHA, the hybrid composite exhibits a stiffness of 7.13 ± 0.12 GPa, tensile strength of 64.48 ± 8.51 MPa and tensile elongation of 1.74 ± 0.58%. Thus hybridizing bioactive nHA with MWNTs can produce PEEK-based hybrid with elastic modulus close to that of cortical bone. Figure 26a shows that the inclusions of 1.5 wt% and 3.0 wt% MWNT to hydrophobic PEEK have little effect in reducing the water contact angles. However, the additions of 1.5 wt% and 3.0 wt% MWNT to PEEK/30 wt% nHA composite lead to a large drop in water contact angle values. The water contact angle declines markedly to 84.08° and 76.99° by hybridizing 30 wt%nHA with 1.5 wt% MWNT and 3.0 wt% MWNT. So a synergistic effect exists between nHA and MWNT in converting hydrophobic PEEK to hydrophilic. Due to improved surface wettability, hydrophilic PEEK/nHA-MWNT hybrids favor cellular adhesion. This is manifested by enhanced proliferation, differentiation and mineralization of murine MC3T3-E1 cells on the hybrid composite surfaces as revealed by MTT, ALP activity and Alizarin red-S staining results (Figure 26b–d). The large increase in ALP activity level of PEEK/30 wt%nHA-1.5 wt% MWNT and PEEK/30 wt%nHA-3.0 wt%MWNT hybrids at day 7 shows good indication of osteoblastic differentiation. 

Nowadays, the emergence and increasing use of nanomaterials have raised the concerns about their potential health hazards. CNTs have emerged as a new tool for transporting and delivering drug molecules [201]. However, CNTs with large aspect ratios and surface areas as well as needle-like features would cause damages to human organs such as skins, lungs, kidneys, brains, etc. Possible entry routes include dermal contact, inhalation, injection and digestion during manufacturing or biomedical use. In vitro and in vivo studies have reported that CNTs can induce cytotoxicity via the generation of ROS and oxidative stress [202,203,204]. From in vivo animal models, needle-like CNTs are trapped by the lung, liver, spleen, etc. upon exposure to nanotubes. CNTs then penetrate the cell wall and reside in the cytoplasm of target organ cells [205]. It is noted those studies were carried out under a direct contact of cell lines or animals with standalone carbon nanotubes. In polymer nanocomposites, CNT fillers are encapsulated and embedded firmly in the polymer matrix, thereby diminishing their toxic effects [61,62,63]. 

### 5.2. PEEK-nHA-CF Hybrids

**Figure 27:**
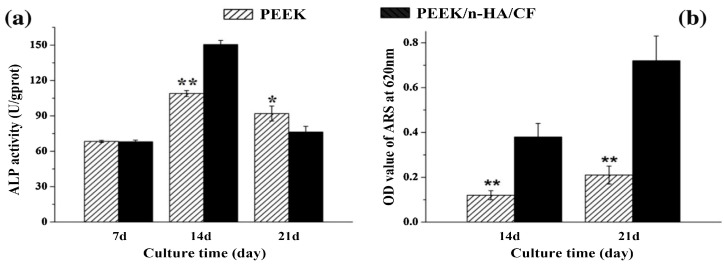

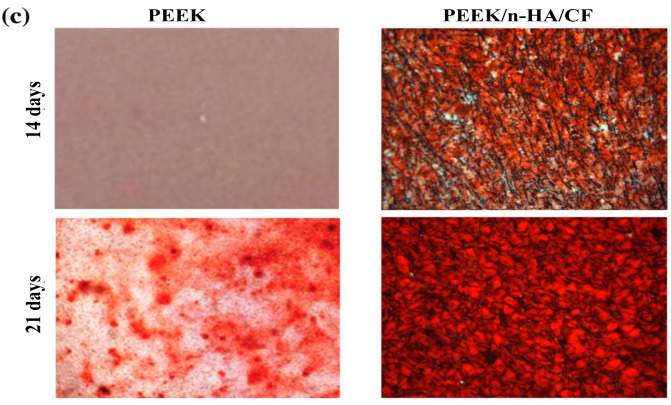
(**a**) ALP activity normalized to protein content of MG63 cells cultured on pure PEEK and PEEK/nHA-SCF hybrid for different time points. (**b**) The quantitative value of matrix mineralization acquired by measuring optical density. (**c**) Alizarin red staining for calcium-rich deposits secreted by MG63 cells on PEEK and PEEK/nHA-SCF hybrid. * represents *p* < 0.05 and ** indicates *p* < 0.01 compared with PEEK/nHA-SCF. Reproduced from [200] with permission of Elsevier.

As mentioned, the tensile properties of injection molded PEEK/30 wt% SCF composite are similar to those of cortical bone [151]. This microcomposite exhibits a poor ability to induce osteoblastic cell adhesion and proliferation. The biocompatibility of PEEK/CF composite can be improved by plasma spraying of nHA on its surface. However, the high-temperature environment of plasma sprayed gun would degrade nHA during deposition [107]. Alternatively, nHA can be used as a bioactive filler for PEEK/CF composites. Recently, Wei and coworkers fabricated PEEK/25wt%nHA–20 wt%SCF via injection molding. The length and diameter of SCFs were 109 µm and 4 µm, respectively. The elastic modulus of PEEK was 3.5 ± 0.3 GPa, and reached 16.5 ± 0.7 GPa by adding 25 wt%nHA and 20 wt%SCF [200]. The addition of 25 wt%nHA to PEEK/20 wt%SCF promotes the adhesion and growth of MG63 osteoblasts on its surface. After proliferation, the cells begin to differentiate by secreting bone matrix proteins. Figure 27a shows the ALP activity of MG63 cells cultured on PEEK/25 wt%nHA–20 wt%SCF for different time points. At day 7, pure PEEK and hybrid composite show a similar ALP activity level. At day 17, the ALP activity level of both samples increases in which the hybrid showing a higher activity. Meanwhile, MG63 cells begin to mineralize the ECM at this stage (Figure 27b). The ALP activity declines on both samples at day 21, which marks the final phase of differentiation. At this time point, the mineralization level on the hybrid is increased largely compared to pure PEEK. Figure 27c shows the formation of mineralized nodules due to osteoblasts on both sample surfaces at day 14 and day 21 as revealed by Alizarin red staining. These results demonstrate that the nHA filler of hybrid composite enhances the mineralization of ECM, thus facilitating the bone formation. In this respect, PEEK/25 wt%nHA–20 wt%SCF hybrid implants are inserted into tooth defects of male beagle dogs by extracting the rear molars in the upper and lower jaws. The hybrid composite implant exhibits higher osseointegration than PEEK based on 3D microcomputed tomography and histological analysis of bone ingrowth in the implants. So, the PEEK/25 wt%nHA–20 wt%SCF hybrid shows potential application for fabricating dental implants.

## 6. Scaffolds for Soft Tissue Engineering

Synthetic polymer scaffolds have been employed in tissue engineering to offer a structurally stable environment for bone regeneration. A scaffold serves as an artificial ECM that mimics the structure and function of natural bone. So, the scaffold should possess a 3D, interconnected porous structure that provides a structural support for the adhesion, growth and differentiation of osteoblasts. As such, the scaffold for bone tissue engineering should provide osteoinductive, osteoconductive, and osteointegrative properties [206,207,208,209]. Human bone tissues comprise of dense cortical bone and porous cancellous/trabecular bone having a porosity of 50–90%. The design of polymer scaffolds concentrates on mimicking cancellous bone structure. The porosity of scaffolds is designed to make them resemble cancellous bone for tissue regeneration. Biodegradable natural and synthetic polymers are typical materials used for constructing porous scaffolds [210,211,212].

Porous polymer scaffolds can be fabricated from several techniques such as solvent casting-particulate leaching, gas foaming, freeze drying, thermally induced phase separation, electrospinning and 3D printing. These techniques have their own advantages and shortcomings [207]. For instance, solvent casting/particulate leaching method involves an initial dissolution of polymer in an organic solvent, followed by porogens mixing. The last step procedures include mold casting and water immersion for leaching porogens. The main limitations of this technique include the use of a toxic solvent for the polymer dissolution, prolonged water soaking, and a small membrane thickness. Electrospinning can produce ECM-mimicking fibrous network with high porosity and interconnected pores. However, the pores generated are rather small, thus preventing the ingrowth of bone cells. In recent years, 3D-printing techniques such as FFF, stereolithography (SLA) and selective laser sintering (SLS) have been used increasingly for printing 3D porous scaffolds for bone tissue engineering [207,213,214,215,216,217]. AM technology is very effective to fabricate porous polymer scaffolds with the accurate control of the pore size, porosity and interconnected pore network. Thus, the printed 3D scaffolds are capable of sustaining vascularization, cell transport and tissue ingrowth [218]. 

**Figure 28:**
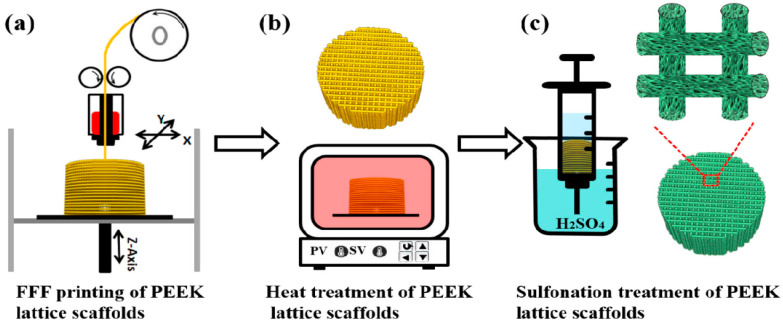
Schematic illustration showing micropores formation on the filaments of FFF-printed PEEK lattice scaffolds. (**a**) FFF printing, (**b**) heat treatment, and (**c**) sulfonation of PEEK scaffolds. Reproduced from [216] with permission of Elsevier under a Creative Commons license.

**Figure 29:**
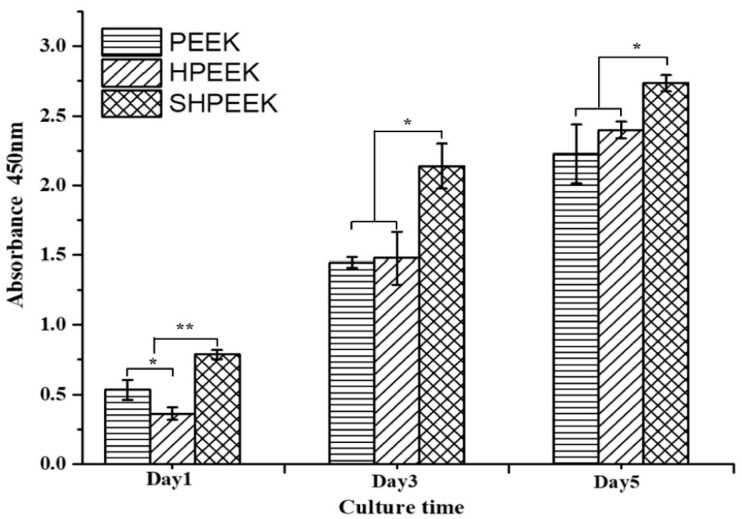
The proliferation of MC3T3-E1 cells on PEEK, HPEEK and SHPEEK scaffolds at different time points. * *p* < 0.05 and ** *p* < 0.01. Reproduced from [216] with permission of Elsevier under a Creative Commons license.

**Figure 30:**
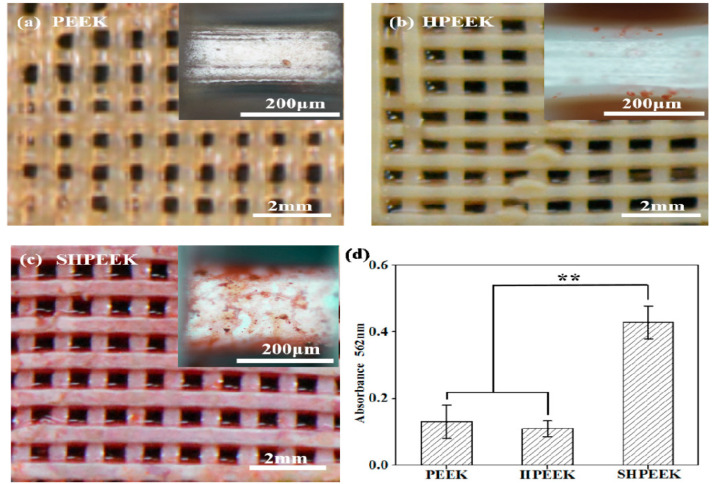
Alizarin red S staining of calcified nodules secreted by MC3T3-E1 cells on different scaffolds at day 7. Optical photographs of Alizarin red S staining of MC3T3-E1 cells on (**a**) PEEK, (**b**) HPEEK, and (**c**) SHPEEK scaffolds. (**d**) Quantitative analysis of deposited calcified nodules. (** *p* < 0.01). Reproduced from [216] with permission of Elsevier under a Creative Commons license.

PEEK is a semicrystalline polymer with a high melting temperature of 340 °C [27]. Because of its high melting temperature, it is more difficult to print 3D PEEK products than other thermoplastics. Nevertheless, some workers have successfully printed porous PEEK scaffolds by monitoring the printing parameters properly [215,216,217]. Very recently, Su et al. employed FFF technique to print 3D porous scaffolds followed by heat treatment and sulfonation. The FFF-printed PEEK scaffolds were heat treated at 200 °C for 1.5 h and then at 300 °C for another 1.5 h to improve the mechanical properties. Subsequently, micropores were induced on the PEEK filaments of heat-treated scaffolds via sulfonation in concentrated sulfuric acid (Figure 28) [216]. Accordingly, bone cells can attach readily onto rough PEEK filaments prior to cellular ingrowth through the large pores created by FFF. Figure 29 shows the proliferation of MC3T3-E1 cells cultured on pure PEEK, heat-treated PEEK (HPEEK) and sulfonated PEEK (SHPEEK) scaffolds for different time points as determined by Cell Counting Kit-8 assay. At day 3, there exists no significant difference in the cell viability between PEEK and HPEEK scaffolds. In contrast, the presence of uniform micropores on SHPEEK scaffold promotes better cellular adhesion and proliferation after culturing for 3 and 5 days. Figure 30a–d show the mineralization of ECM by MC3T3-E1 cells cultured on different scaffolds for 7days. Very few calcified nodules are deposited on the surface filaments of PEEK and HPEEK scaffolds. However, the filaments of SHPEEK scaffold are completely stained with a red dye, demonstrating the abundant deposition of calcified nodules on the filaments. Thus, micropores created on the PEEK filaments provides the sites for osteoblastic adhesion, proliferation and mineralization.

### Biodegradable PEEK Blend Scaffolds

The use of PEEK as a biomaterial to make scaffolds in soft tissue engineering is hampered by its non-degradation behavior. PEEK is hydrophobic, exhibiting no degradation upon exposure in aqueous environments. On the other hand, biodegradable polymers such as polyglycolic acid (PGA) and polyvinyl alcohol (PVA) can be blended with PEEK to produce degradable scaffolds [219,220,221]. PGA is an aliphatic polyester showing very fast degradation in aqueous solutions due to its hydrolytic nature. PGA decomposes into glycolic acid through ester hydrolysis. The acidic product further degrades the polymer in an autocatalytic effect [222]. PGA is often used as a suture material clinically. However, the fast degradation rate and poor mechanical strength limit its application in tissue engineering [223]. By combining PEEK with PGA, the resulting PEEK/PGA blends are able to degrade; the degradation rate can be tuned by monitoring the PGA content of PEEK/PGA blends. In addition, PEEK with high stiffness and strength can sustain the structure of PEEK/PGA scaffolds from collapsing during the degradation of PGA in aqueous environments. 

**Figure 31:**
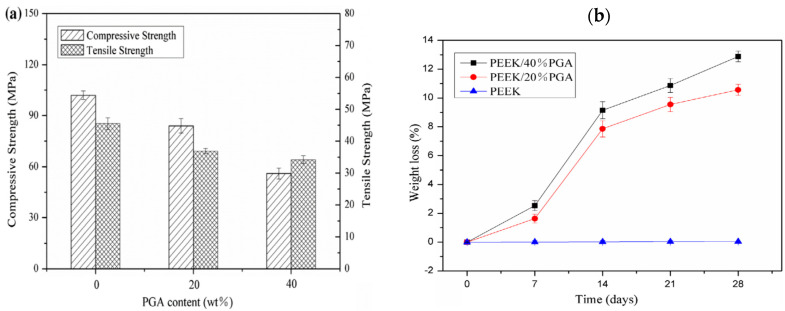
(**a**) Mechanical strength vs PGA content of SLS-printed PEEK and PEEK/PGA blend scaffolds. (**b**) Weight loss vs time plots of SLS-printed PEEK and PEEK/PGA blend scaffolds immersed in a simulated body fluid at 37 °C for different time periods. Reproduced from [219] with permission of Taylor & Francis.

More recently, Shuai et al. fabricated porous PEEK/PGA blend scaffolds containing 20 wt% and 40 wt% PGA using selective laser sintering (SLS) process [219]. SLS is one of the AM techniques employing a laser beam to sinter polymer powder materials in a layer by layer to construct 3D scaffolds. Figure 31a shows the compressive strength and tensile strength vs PGA content for SLS-printed PEEK/PGA scaffolds. Both mechanical strengths decrease with increasing PGA content as expected. The compressive strength of printed PEEK and PEEK/PGA scaffolds is higher than that of cancellous bone ranging from 2–12 MPa [224]. Figure 31b shows the weight loss vs time plots of PEEK, PEEK/20 wt% PGA and PEEK/4 0wt% PGA scaffolds upon immersion in a simulated body fluid (SBF) at 37 °C for different periods. The PEEK scaffold exhibits no weight loss after immersion in SBF for up to 28 days. However, PEEK/PGA scaffolds show distinct degradation behavior in SBF as revealed by a marked increase in the weight loss with immersion time. The weight loss of the scaffolds increases with increasing PGA content in the polymer blend. The water uptake into hydrolytic PGA phase leads to its dissolution in SBF. Consequently, degradation-induced pores are found on the surfaces of PEEK/PGA blend scaffolds. 

**Figure 32:**
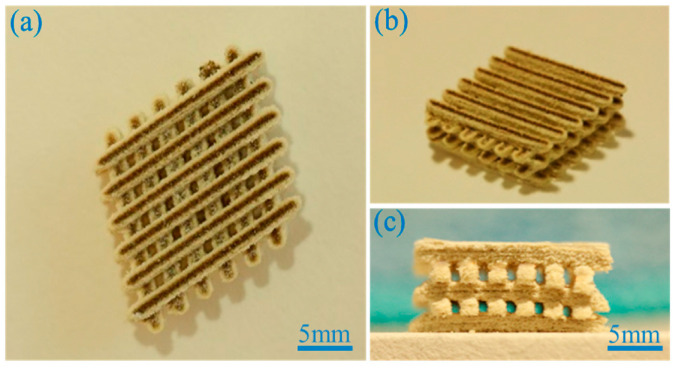
(**a**) Inclined, (**b**) top, and (**c**) lateral view images of SLS-printed PEEK-20 wt% PGA/nHA scaffold. Reproduced from [220] with permission of MDPI under the terms of the Creative Commons Attribution license.

**Figure 33:**
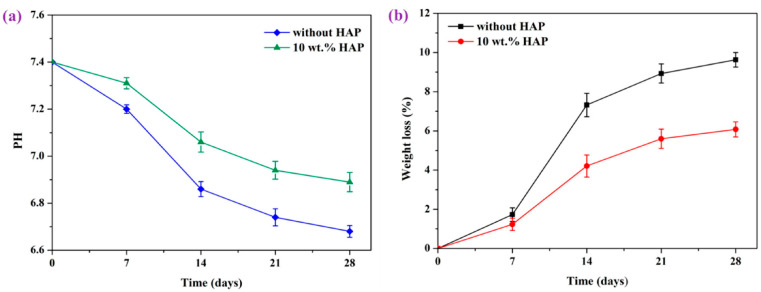
The plots of (**a**) pH value vs time, and (**b**) weight loss vs time of PEEK-20 wt%PGA and PEEK-20 wt%PGA/10 wt% nHA scaffolds upon immersion in PBS at 37 °C. Reproduced from [220] with permission of MDPI under the terms and conditions of the Creative Commons Attribution license.

**Figure 34:**
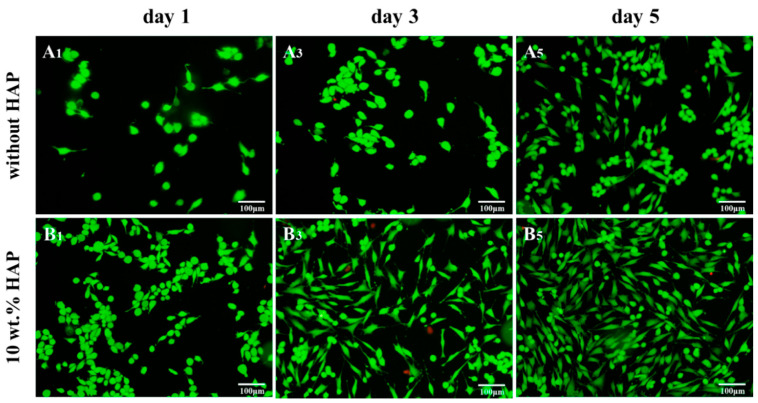
Fluorescence images of MG63 cells cultured on PEEK-20 wt% PGA (top panel) and PEEK-20 wt%PGA/10 wt% nHA (bottom panel) scaffolds for different time periods. HAP: nanohydroxyapatite. Scale bar: 100 µm. Reproduced from [220] with permission of MDPI under the terms and conditions of the Creative Commons Attribution license.

PEEK and PEEK-PGA scaffolds are bioinert, thus their surfaces are unfavorable for osteoblastic adhesion and proliferation. The scaffolds must achieve good compatibility with the surrounding tissue to ensure bone regeneration. In general, SLS-built scaffolds have rough surfaces, contributing somewhat to bone cell adhesion. So sulfonation is unnecessary for creating micropores on the PEEK filaments of scaffolds for cell attachment. The biocompatibility of SLS-printed PEEK and PEEK-PGA scaffolds can be further improved by adding nHA fillers. In another study, Shuai et al. incorporated 5–15 wt% nHA into PEEK-20 wt% PGA scaffold. Figure 32a–c are the photographs showing typical features of SLS-printed PEEK-20%PGA/nHA scaffold viewed at different directions. The degradation behaviors of PEEK-20%PGA and PEEK-20%PGA/10%nHA scaffolds in phosphate buffer solution (PBS) with an initial pH of 7.4 at 37 °C are depicted in Figure 33a,b. The pH of PBS decreases with immersion time due to the release of glycolic acid byproduct. The 10%nHA filler of the PEEK-20%PGA/10%nHA scaffold neutralizes autocatalytic effect due to acidic PGA byproduct. This is beneficial in reducing inflammation of wounds. So, PEEK-20%PGA scaffold exhibits a higher weight loss at different time points compared to PEEK-20%PGA/10%nHA due to the autocatalytic mechanism [222]. The nHA filler favors the adhesion and proliferation of MG63 cells on the surface of PEEK-20%PGA/10%nHA scaffold (Figure 34). The [3-(4,5-dimethylthiazol-2-yl)-2,5-diphenyltetrazolium bromide] MTT cell proliferation assay results also reveal that the cell viability of PEEK-20%PGA/10%nHA is higher than that of PEEK-20%PGA. Finally, ALP activity level of MG63 cells cultured on the PEEK-20%PGA/10%nHA scaffold is considerably higher than that on PEEK-20%PGA scaffold.

**Figure 35:**
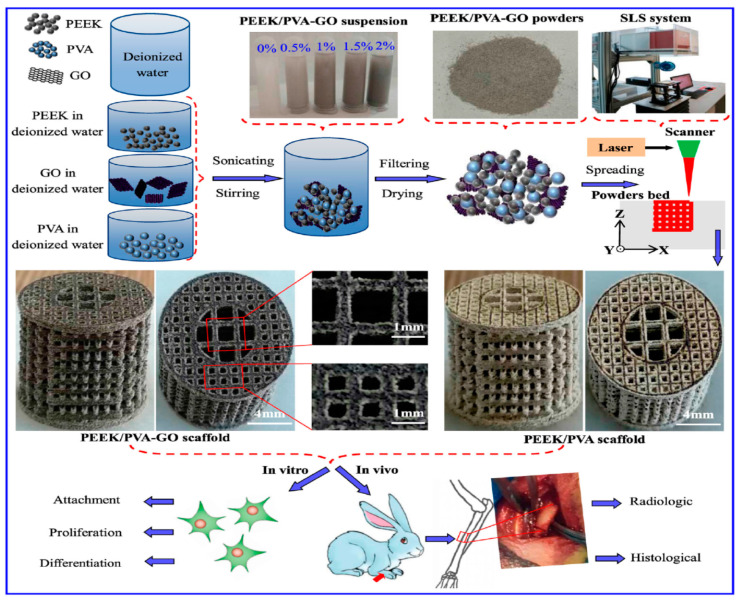
Schematic illustration showing the fabrication of porous PEEK-PVA and PEEK-PVA/GO scaffolds through solution mixing and selective laser sintering. These scaffolds are subjected to in vitro cell culture and in vivo animal model studies. Reproduced from [221] with permission of Taylor & Francis.

As mentioned above, GO additions show little enhancement in stiffness of injection molded PEEK/GO nanocomposites [197]. On the contrary, GO sheets can be dispersed homogeneously in the polymeric matrix using solution mixing process. In this respect, hydrolytic PVA, GO and PEEK are dispersed and mixed in water under sonication, followed by filtering and drying. The laser beam of SLS is then used to sinter mixed PEEK-PVA/GO powders to form 3D porous scaffolds. The mass ratio of PEEK to PVA is fixed at 7:3 (*wt/wt*) (Figure 35) [221]. 

**Figure 36:**
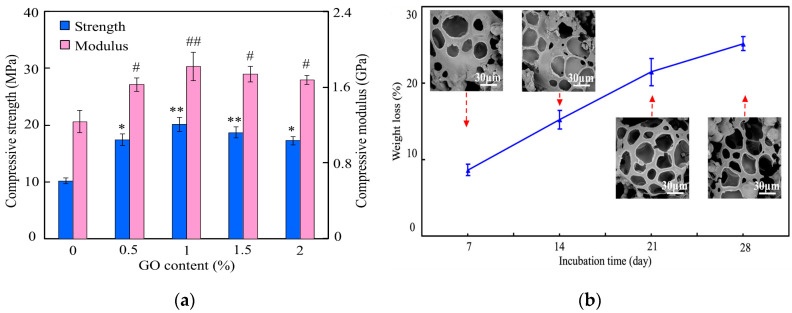
(**a**) Compressive strength and compressive modulus of porous PEEK-PVA scaffolds as a function of GO content. # denotes statistically significant difference between compressive strength of the scaffolds (*p* < 0.05), * denotes statistically significant difference between compressive modulus of the scaffolds (*p* < 0.05). (**b**) Weight loss vs time of PEEK-PVA/1 wt% GO scaffold upon immersion in PBS at 37 °C for 28 days. Reproduced from [221] with permission of Taylor & Francis.

The additions of 0.5–2 wt% GO to PEEK-PVA blend enhance the compressive strength and compressive modules markedly, especially at 1wt% GO (Figure 36a). These results reveal remarkable strengthening and stiffening effects of GO nanofillers. The compressive strength and compressive modulus of PEEK-PVA scaffold are determined to be 10.12 MPa and 1.22 GPa, respectively; while those of PEEK-PVA/1 wt% GO scaffold are 20.13 MPa and 1.82 GPa, respectively. So, the compressive strength of PEEK-PVA/1 wt% GO scaffold is twice higher than that of PEEK-PVA scaffold. Furthermore, the compressive strength of PEEK-PVA/1 wt% GO scaffold is considerably higher than that of cancellous bone ranging from 2–12 MPa [224]. From the literature, the compressive modulus of wet human cancellous bone and dry bovine cancellous bone is 0.44 ± 0.27 GPa and 1.4 ± 0.3 GPa, respectively [225]. The compressive modulus of PEEK-PVA/1 wt% GO scaffold exceeds that of mammalian cancellous bone. So, this composite scaffold with enhanced compressive strength and stiffness can provide a strong support for bone cell adhesion, proliferation and bone tissue regeneration. It is well known that GO is hydrophilic due to the presence of oxygenated functional groups. PEEK is hydrophobic, and the water contact angle reduces to 85.52° by blending with hydrolytic PVA. The water contact angle further reduces to 78.16° by adding 1wt% GO to PEEK-PVA. As a result, the water absorption of PEEK-PVA/1wt% GO scaffold increases with immersion time in PBS at 37 °C, leading to the dissolution of PVA phase and weight loss increment accordingly. Some pores are formed on the scaffold surface due to the PVA degradation, and the number of pores increases with immersion time (Figure 36b). The GO sheets are known to promote the adhesion, growth and differentiation of osteoblasts [76,226,227]. Therefore, PEEK-PVA/1 wt% GO scaffold promotes the attachment, proliferation and differentiation of MG63 cells as revealed by live/dead assay and CCK-8 assay results and encourages new bone formation in New Zealand white rabbits in vivo. Table 3 lists the strengths and weaknesses of 3D-printed PEEK-based scaffolds prepared by additive manufacturing process. Table 4 summarizes the compression mechanical behaviors, in vitro and in vivo animal test properties of these scaffolds.

In general, the translation of newly developed scaffolds from the laboratories to clinical trials involves a long-term period of intense research and testing. It depends on the successful completion of a series of technical requirements and measurements including in vitro cell culture tests, biomechanical measurements for assessing BIC, in vivo animal models for evaluating the repair potential of bone scaffolds, and ethical approval for human trials. In terms of in vitro cell culture tests, several cell types such as osteoblasts, fibroblasts and hMSCs are needed for assessing cell adhesion, growth and differentiation on the scaffolds. The hMSCs cultivation is particularly useful because they can differentiate to osteoblastic lineages on the scaffolds by expressing osteogenic marker genes such as COL1, ALP, osteopontin and osteocalcin [229]. The development of 3D nondegradable PEEK and degradable PEEK scaffolds for bone tissue has attracted attention in materials community very recently. At present, the researchers have only cultivated murine MC3T3-E1 and human MG63 cells on those scaffolds [216,219,220,221]. No works have been reported in the literature on the differentiation of hMSCs on those scaffolds. For in vivo animal models, the repair ability and bone formation of newly developed scaffolds in different animal species, including mouse, rabbit, goat, dog, etc., can be assessed. Therefore, proper selection of an animal model, location of bone defect for scaffold treatment, and the age and health condition of the model should be taken into consideration [230]. Till to present, only one rabbit model has been reported relating new bone formation in the PEEK-PVA/1%GO scaffold [221]. Based on these literature reports, more MSCs cell culture and in vivo animal model tests are needed to assess the repair ability, safety and efficacy of PEEK-based scaffolds prior to human trials. Thanks to the wide applications of PEEK in spinal fusion devices as mentioned above, the information relating clinical trials of PEEK spinal fusion devices can be obtained accordingly. Such information is particularly useful for reducing the clinical trial periods and unnecessary biological testing of PEEK-based scaffolds. 

## 7. Future Prospects and Challenges

The majority of PEEK implants for orthopedic applications are cervical and lumbar spinal cages. In particular, surface porous PEEK implants prepared by melt-extrusion and porogen leaching can generate rough surfaces for osteoblastic cell adhesion, growth and differentiation, thereby facilitating bone ingrowth. Ti-coated PEEK implants prepared by plasma spraying also exhibit rough surfaces, acting as the sites for bone cell adhesion and proliferation as well as upregulated ALP activity and BMP-2 levels [116]. Those implants exhibit large pullout force as revealed by biomechanical pullout tests, resulting from mechanical interlocking of bone with the rough coating surface. The enhanced osteogenic differentiation in vitro and large pullout force in vivo lead to the implementation of Ti-PEEK implants for posterior lumbar interbody fusion (PLIF) surgery [121]. However, such implants suffer from the delamination and wear of metallic Ti layer [120]. The delamination of Ti coating could cause implant loosening. The wear debris of Ti particles would induce an immune response and chronic inflammation. Therefore, many challenges remain in solving the wear issues of Ti-PEEK implants. Unless the wear problem is solved, one would prefer the use of surface porous PEEK implants with enhanced osseointegration than Ti-PEEK for spinal fusion surgery. 

The mechanical strength of PEEK/mHA microcomposites is insufficient for load bearing applications due to debonding of mHA fillers from the PEEK matrix. The tensile strength of these microcomposites decreases with increasing mHA content. Silane coupling agents cannot be used for enhancing the filler-matrix interactions due to adverse health effects. In this respect, nHA fillers are incorporated into PEEK to form PEEK/nHA nanocomposites. From Table 1 and Table 2, the stiffness of PEEK can be increased to 6.20 ± 0.13 GPa and 7.85 ± 0.11 GPa by adding 30 wt% and 40 wt% nHA, respectively. The PEEK/40 wt% nHA with a tensile elongation of 0.69 ± 0.21% is very brittle. Thus, a balance must be maintained between the stiffness and ductility in the design of biocomposites. As such, low MWNT contents (i.e., 1.5 and 3 wt%) are added to PEEK/30 wt% nHA composite to form hybrids. The PEEK/30 wt% nHA–3.0 wt% MWNT hybrid with a stiffness of 7.13 ± 0.12 GPa, tensile strength of 64.48 ± 8.51 MPa and fracture elongation of 1.74 ± 0.58% show similar tensile properties with those of cortical bone. 

From the perspective of health, the potential risks from exposure to MWNTs must be assessed [231]. Considering nanometer dimension of MWNTs, they can penetrate easily into human cells through several ways including skin penetration, inhalation, injection and ingestion. MWNTs contain catalytic metal nanoparticles (e.g., Fe, Ni, Co, etc.), which are needed for the synthesis of nanotubes [232]. Those metal catalysts together with needle-like feature of nanotubes would induce ROS and oxidative stress, leading to apoptosis of several human cell types [231,233]. For the PEEK/30 wt%nHA–3.0 wt% MWNT hybrid, the toxic effects of MWNTs are minimized because they are embedded firmly in the PEEK matrix. On the other hand, GO sheets without metal catalysts are considered as an alternative reinforcing material [76]. However, low loading levels of GOs are rather difficult to disperse homogenously in the PEEK matrix during melt compounding and injection molding. Little enhancement in stiffness is achieved in injection molded PEEK/(0.1–5 wt%)GO nanocomposites as mentioned previously [197]. More studies relating the fabrication, in vitro cell culture and in vivo animal model of injection molded PEEK/nHA-GO are needed in near future to ensure their suitably for making load-bearing implants. 

It remains a great challenge for the researchers to design load-bearing hip implants. The long-term success of artificial femoral stems primarily depends on their safe integration into the host tissues. PEEK/CF and PEEK/SCF composites are particularly suitable for load-bearing hip stems due to the stiffening and strengthening effects of carbon fibers. In particular, the tensile properties of PEEK/30 wt% SCF composite resemble those of cortical bone. This composite also possesses favorable wear resistant properties on a hip simulator for 10 million cycles. The biocompatibility of PEEK/SCF composites can be improved by adding nHA [206]. In vitro and in vivo animal model results reveal that PEEK/SCF-nHA hybrid facilitates MG63 cell attachment, proliferation and differentiation as well as osseointegration. Till to present, Ti-6Al-4V alloy is still used primarily for fabricating load-bearing femoral stems. Ti-6Al-4V alloy contains toxic V element and shows stress-shielding effect. Thus PEEK/CF and PEEK/SCF composites with mechanical stiffness and strength close to cortical bone show a great potential for replacing Ti–6Al–4V alloy in load-bearing implants. However, clinically available PEEK/CF and PEEK/SCF composites with nHA coating/filler for femoral stems have been very slow. The in vivo data relating the PEEK/CF composites for hip stem applications are very scarce in the literature. The HA-coated PEEK/CF hip stem implant in an ovine model exhibits bone on-growth fixation and minimal stress shielding [147,148]. So, many efforts and further studies are needed for the development of biocompatible PEEK/CF and PEEK/SCF composites for hip stem applications. 

Microbial contamination on hip implants, dental and spinal fusion devices is an unresolved issue that leads to the failure of implants, infection, morbidity and mortality [170,234,235]. Little information is available in the literature relating antibacterial activity of PEEK/SCF composites and PEEK/nHA nanocomposites. With the advent of nanotechnology, novel nanoparticles with bactericidal activity have been developed for biomedical applications. Wang et al. demonstrated that PEEK/40 wt% nano-FHA composite exhibits superior resistance against *S. mutans* [164]. Díez-Pascual et al. reported that PEEK/ZnO nanocomposites show good bactericidal activity against *S. aureus* and *E. coli* [179,182]. ZnO nanoparticles can generate ROS under UV and visible light irradiation, and even in the dark for bacterial inactivation. Furthermore, ZnO NPs can release Zn^2+^ ions upon direct contact with bacteria strains, thereby disrupting their cell membranes. However, ZnO NPs may have hazardous health effects. The risks from nanomaterials arise from their unknown adverse effects and properties [236]. There are no reports relating in-vivo animal models of PEEK/ZnO nanocomposites. Further animal models are required to confirm the successful clinical implementation of those nanocomposite implants with antibacterial activity. 

## 8. Conclusions

Metallic implants made from titanium alloy, cobalt chrome molybdenum alloy and austenitic stainless steel with high elastic modulus have noted complications associated with stress shielding, corrosion and cytotoxic effects of released metallic ions. Thus, PEEK with a lower modulus than metals is considered as a material substitute for metallic implants. However, hydrophobic PEEK is bioinert, showing poor osteointegration with bone tissues after implantation. The bioactivity of PEEK can be largely improved by either surface modification, coating deposition, or mHA/nHA filler addition. The former strategy includes the creation of surface porous layer on the medical implant. By combining melt extrusion and porogen leaching process, porous PEEK surface layer with a porosity of 67.3% has been developed for use in anterior cervical discectomy and fusion (ACDF) surgery (COHERE^®^) [97]. Otherwise, the deposition of HA, Ti, or TiO_2_ coating layer on PEEK by means of vacuum plasma spraying, aerosol deposition, cold spraying, magnetron sputtering, etc. can also improve osseointegration performance. However, plasma spraying of HA on PEEK would lead to inhomogeneous melting of HA into tricalcium and tetracalcium phosphates, degrading the performance of HA coating accordingly. So, aerosol deposition and cold spraying are adopted for coating HA on PEEK to resolve this issue. Meanwhile, plasma spraying of Ti coating on PEEK can produce a rough surface that facilitates the adhesion and growth of osteoblasts. As such, Ti-PEEK cages have been developed for posterior lumbar interbody fusion, with 24 patients participate in a clinical trial for fusion implants [121].

The elastic modulus of PEEK (3.7–4.0 GPa) is considerably lower than that of cortical bone ranging from 7 to 30 GPa. Thus, pure PEEK is not stiff enough to sustain applied stress for load-bearing implants. The modulus of PEEK can be enhanced by adding HA fillers or carbon fibers. The HA with a modulus of 120.6 GPa can stiffen PEEK [26]. However, it requires more than 30 wt% mHA to achieve desired tensile properties and biocompatibility [135]. Due to poor interfacial bonding, large mHA particulates often debond from the PEEK matrix during tensile testing. This leads to resulting composites with low mechanical strength. In this respect, carbon fibers with a stiffness ≥ 230 GPa are used to stiffen PEEK. In particular, discontinuous carbon fibers can be readily melt-compounded with PEEK in conventional extruders and injection molders for forming composites. The stiffness of PEEK/30 wt% SCF composite reaches as high as 18.5 ± 2.3 GPa [151], while that of PEEK/5wt% SCF composite reaches 7.37 ± 1.22 GPa. The stiffness and tensile strength of PEEK/5 wt% SCF and PEEK/30 wt% SCF composites match closely with those of cortical bone. However, PEEK/SCF composites are bioinert, showing poor osteoblastic adhesion and proliferation. By adding 25 wt% nHA to PEEK/20 wt% SCF, the resulting hybrid composite has a stiffness of 16.5 ± 0.7 GPa with good biocompatibility [206]. Thus, the mechanical properties and biocompatibility of PEEK/SCF-nHA hybrids can be tailored by adjusting SCF and nHA contents. 

Hydrophobic PEEK shows non-degradation behavior in aqueous solutions, thus hampering its use as a scaffolding material for soft tissue engineering. The degradation of PEEK can be improved by blending with PGA and PVA. In this respect, porous PEEK blend scaffolds prepared by AM process exhibit apparent degradation in SBF and PBS solutions due to the dissolution of hydrolytic polymers. The PEEK blend scaffolds exhibit good compressive strength to support the structure of scaffolds from collapsing during the degradation of hydrolytic polymers. Finally, the biocompatibility of porous PEEK blend scaffolds can be greatly enhanced by adding nHA or GO. 

## Figures and Tables

**Table 1:** Tensile properties of pure PEEK and its microcomposites.

Sample	Elastic Modulus, GPa	Tensile Strength, MPa	Elongation at Break,%	Reference
Pure PEEK	3.90 ± 0.2	93 ± 1	66 ± 7	[150]
Pure PEEK	3.87 ± 0.10	80.06±0.49	67.10 ± 13.40	[62,63]
Pure PEEK	3.79 ± 0.27	95.21 ± 1.86	N.A.	[45]
Pure PEEK	2.20 ± 0.17	84.0±1.9	N.A.	[87]
PEEK/10 vol% (20.79 wt%) mHA	4.33 ± 0.94	64.71±1.46	19.23 ± 2.74	[131]
PEEK/20 vol% (37.64 wt%) mHA	4.78 ± 1.38	58.59 ±1.91	4.26 ± 0.72	[131]
PEEK/30 vol% (50.86 wt%) mHA	8.17 ± 1.09	49.15 ±2.08	1.96 ± 0.08	[131]
PEEK/40vol% (61.68wt%) mHA	15.37 ± 3.30	43.76 ±5.26	0.98 ± 0.52	[131]
PEEK/30 wt% mHA	7.2	57	N.A.	[135]
PEEK/40 wt% mHA	10.4	45 ± 2.5	N.A.	[135]
Endolign^®^ (unidirectional)	150	2000	N.A.	[146]
Endolign^®^ (multidirectional)	70	900	N.A.	[146]
PEEK/5 wt% SCF	7.37 ± 1.22	101.41 ± 4.23	N.A.	[45]
PEEK/30 wt% SCF (PAN)	24	214	2.0	[42]
PEEK/30 wt% SCF (PAN)	18.5 ± 2.3	192 ± 17	1.9 ± 0.2	[150]
PEEK/30 wt% SCF (Pitch)	12.5 ± 1.3	145 ± 9	2.2 ± 0.2	[150]
Human cortical bone	7–30	50–100	1–3	[9]

N.A.: Not available.

**Table 2:** Tensile properties of PEEK-based nanocomposites and hybrids.

Sample	Elastic Modulus, GPa	Tensile Strength, MPa	Elongation at Break, %	Ref.
*PEEK-based Hybrid Composites*:				
PEEK/30 wt% nHA-2 wt% CNF	6.54 ± 0.29	71.67 ± 5.25	2.83 ± 0.66	[62]
PEEK/30 wt% nHA-1.5 wt% MWNT	6.83 ± 0.20	70.99 ± 7.51	2.32 ± 1.15	[63]
PEEK/30 wt% nHA-3.0 wt% MWNT	7.13 ± 0.12	64.48± 8.51	1.74 ± 0.58	[63]
PEEK/25 wt% nHA-20 wt% SCF	16.5 ± 0.7	138	N.A.	[200]
*PEEK-based Nanocomposites*:				
PEEK/ 10wt% nHA	4.34 ± 0.08	80.55 ± 0.15	31.4 ± 5.18	[62]
PEEK/20 wt% nHA	4.92 ± 0.06	81.23 ± 0.55	7.62 ± 0.27	[62]
PEEK/30 wt% nHA	6.20 ± 0.13	70.56 ± 3.22	2.71 ± 0.34	[62]
PEEK/40 wt% nHA	7.85 ± 0.11	44.51 ± 7.53	0.69 ± 0.21	[62]
PEEK/20 wt% nHA	3.40 ± 0.20	81.0 ± 2.4	N.A.	[87]
PEEK/40 wt% nHA	4.60 ± 0.12	75.0 ± 2.7	N.A.	[87]
PEEK/1.5 wt% MWNT	4.21 ± 0.11	83.38 ± 0.78	57.25 ± 13.20	[63]
PEEK/3.0 wt% MWNT	4.25 ± 0.85	82.08 ± 3.68	56.48 ± 24.90	[63]
PEEK/6.5 wt% MWNT	5.32	102.15	12.49	[198]
PEEK/12 wt% MWNT	6.35	107.14	8.28	[198]
PEEK/15 wt% MWNT	7.55	110.90	6.28	[198]

N.A.: Not Available.

**Table 3:** The strengths and weaknesses of 3D-printed PEEK-based scaffolds.

Scaffold	Strengths	Weaknesses
FFF-printed PEEK	Ease of fabrication. No additional processing steps are needed.	The printed scaffolds are bioinert and nondegradable. No micropores formed on the filaments of printed scaffolds for osteoblastic adhesion. The macro-pores formed between the filaments created by printing are far too large for bone cell adhesion
SHPEEK	Sulfonation of FFF-printed PEEK creates micropores on the filaments for bone cell adhesion	Sulfuric acid residuals can damage bone cells and reduce their viability greatly. After sulfonation, the scaffolds must be rinsed in water several times to remove the residuals until they are contamination-free. So, it is a tedious process.
SLS-printed PEEK-PGA	Degradable scaffolds due to the dissolution of PGA. SLS process creates rough surface needed for bone cell adhesion	High-temperature laser beam used for sintering polymer powders would degrade their properties. Raw polymer powders trap inside the fine voids of scaffolds due to printing are difficult to remove and may induce inflammation [228].
SLS-printed PEEK-PGA/nHA	nHA and rough surface finish are beneficial for bone cell adhesion. nHA neutralizes autocatalytic effect of acidic PGA byproduct, thus reducing inflammation of wounds	As above
SLS-printed PEEK-PGA/GO	GO sheets with high stiffness and strength increase the compressive strength/stiffness of resulting nanocomposite scaffolds. GO promotes bone cell adhesion and growth	GO sheets must be firmly attached in the matrix of composite scaffold. Otherwise, stand-alone or delaminated GO may induce cytotoxicity to human cells [76]

**Table 4:** Compressive mechanical properties, in vitro and in vivo animal test results of 3D-printed PEEK-based scaffolds.

Scaffold	Compressive Strength, MPa	Compressive Modulus, GPa	In Vitro Properties	In Vivo Animal Model	Ref.
SHPEEK	N. A.	N. A.	MC3T3-E1 cells adhere & grow on the micropores created by sulfonation. Mineralization of ECM by bone cells	N. A.	[216]
PEEK-20% PGA	82.5	N. A.	MG63 cells adhere on rough surface of scaffold	N. A.	[219]
PEEK-40% PGA	52.5	N. A.	MG63 cells adhere on rough surface of scaffold	N. A.	[219]
PEEK-20% PGA/10% nHA	92.5	3.31	Adding 10% nHA to PEEK-20%PGA increases cell viability and ALP activity of MG63 cells.	N. A.	[220]
PEEK-PVA	10.12	1.22	PVA improves wettability of PEEK by reducing water contact angle to 85.52°.	N. A.	[221]
PEEK-PVA/1%GO	20.13	1.82	Adding 1% GO to PEEK-PVA promotes the adhesion, growth and differentiation of MG63 cells. GO facilitates the dissolution of PVA phase in PBS solution, & further reduces water contact angle to 78.16°.	GO sheets enhance new bone formation in rabbits	[221]
Cancellous bone	2–14	0.44 ± 0.27	N. A.	N. A.	[224,225]

## Abbreviations

ACDF	Anterior cervical discectomy and fusion
AIP	Arc ion plating
ALP	Alkaline phosphatase
AM	Additive manufacturing
APTMS	Anilinopropyltrimethoxysilane
BIC	Bone implant contact
BMP-2	Bone morphogenetic protein-2
CAD	Computer aided design
CAM	Computer aided manufacturing
CF	Carbon fiber
CLSM	Confocal laser scanning microscopy
CNF	Carbon nanofiber
CNT	Carbon nanotube
COL1	Collagen type 1
CS	Calcium silicate
CT	Computed tomography
ECM	Extracellular matrix
FDM	Fused deposition modeling
FFF	Fused filament fabrication
GO	Graphene oxide
HA	Hydroxyapatite
HDPE	High-density polyethylene
hMSC	Human mesenchymal stem cell
HPEEK	Heat-treated PEEK
IBAD	Ion beam assisted deposition
IPD	Ionic plasma deposition
MC3T3-E1	Murine pre-osteoblastic cell
MG63	Human osteoblast-like osteosarcoma cell
mHA	Hydroxyapatite microparticle
MTT	[3-(4,5-dimethylthiazol-2-yl)-2,5-diphenyltetrazolium bromide]
MWNT	Multiwalled carbon nanotube
nHA	Nanohydroxyapatite
NP	Nanoparticle
OPN	Osteopontin
PAN	Polyacrylonitrile
PBS	Phosphate-buffered saline
PEEK	Polyetheretherketone
PIII	Plasma immersion ion implantation
PGA	Polyglycolic acid
PLIF	Posterior lumbar interbody fusion
PSI	Patient specific implant
PVA	Polyvinyl alcohol
ROS	Reactive oxygen species
SBF	Simulated body fluid
SCF	Short carbon fiber
SHPEEK	Sulfonated PEEK
SLS	Selective laser sintering
SWNT	Single walled carbon nanotube
UHMWPE	Ultra-high molecular weight polyethylene
UV	Ultraviolet
VGCNF	Vapor grown carbon nanofiber
VPS	Vacuum plasma spraying
WST-1	2-(4-iodophenyl)-3-(4-nitrophenyl)-5-(2,4-disulfophenyl)-2H-tetrazolium
YSZ	Yttria-stabilized zirconia
